# Changes in plasma phospholipid fatty acid profiles over 13 years and correlates of change: European Prospective Investigation into Cancer and Nutrition-Norfolk Study

**DOI:** 10.1093/ajcn/nqz030

**Published:** 2019-04-17

**Authors:** Ju-Sheng Zheng, Fumiaki Imamura, Stephen J Sharp, Albert Koulman, Julian L Griffin, Angela A Mulligan, Robert Luben, Kay-Tee Khaw, Nicholas J Wareham, Nita G Forouhi

**Affiliations:** 1 MRC Epidemiology Unit, University of Cambridge, Cambridge, United Kingdom; 2 School of Life Sciences, Westlake University, Hangzhou, China; 3 MRC Elsie Widdowson Laboratory, Cambridge, United Kingdom; 4 NIHR BRC Nutritional Biomarker Laboratory, Cambridge, United Kingdom; 5 Department of Biochemistry, University of Cambridge, Cambridge, United Kingdom; 6 Department of Public Health and Primary Care, University of Cambridge, Cambridge, United Kingdom

**Keywords:** plasma phospholipid fatty acids, saturated fatty acids, trans-fatty acids, n–3 polyunsaturated fatty acids, change, correlates, repeated measurement

## Abstract

**Background:**

Little is known about changes in blood fatty acid compositions over time and the correlates of any changes in a general population.

**Objective:**

The aim of this study was to estimate changes in 27 individual plasma phospholipid fatty acids and fatty acid groups over time, and to identify potential correlates of these changes.

**Methods:**

Plasma phospholipid fatty acids were profiled at 3 time-points (1993–1997, 1998–2000, 2004–2011) among 722 participants in the European Prospective Investigation into Cancer and Nutrition-Norfolk Study, UK. Linear regression models were used to estimate both *1*) mean changes over time in 27 individual fatty acids and 8 prespecified fatty acid groups and *2*) associations of changes in dietary and lifestyle factors with changes in the 8 fatty acid groups, mutually adjusted for dietary/lifestyle factors and other confounders. The prespecified fatty acid groups were odd-chain saturated fatty acids (SFAs), even-chain SFAs, very-long-chain SFAs, marine n–3 polyunsaturated fatty acids (PUFAs), plant n–3 PUFA, n–6 PUFAs, monounsaturated fatty acids (MUFAs), and trans-fatty acids (TFAs).

**Results:**

Adjusted for confounders, fatty acid concentrations decreased for odd-chain SFAs (annual percentage difference in mol percentage: −0.63%), even-chain SFAs (−0.05%), n–6 PUFAs (−0.25%), and TFAs (−7.84%). In contrast, concentrations increased for marine n–3 PUFAs (1.28%) and MUFAs (0.45%), but there were no changes in very-long-chain SFAs or plant n–3 PUFA. Changes in fatty acid levels were associated with consumption of different food groups. For example, a mean 100 g/d increase in fatty fish intake was associated with a 19.3% greater annual increase in marine n–3 PUFAs.

**Conclusions:**

Even-chain SFAs and TFAs declined and marine n–3 PUFAs increased over time. These changes were partially explained by changes in dietary habits, and could potentially help interpret associations of baseline fatty acid composition with future disease risk.

## Introduction

Dietary fat and blood or adipose tissue fatty acids have been associated with the risk of many diseases (1). Blood fatty acids serve in epidemiological studies as objective biomarkers of dietary fat intake, such as the essential PUFAs: linoleic acid (an n–6 PUFA: C18:3n–6) and α-linolenic acid (an n–3 PUFA: C18:3n–3), or odd-chain SFAs (C15:0, C17:0) (2). Some blood fatty acids reflect both dietary source and endogenous biological synthesis, such as even-chain SFAs (C14:0, C16:0, C18:0), MUFAs, and marine n–3 PUFAs (C20:5n–3, C22:5n–3, C22:6n–3), whereas some blood fatty acids, such as C18:3n–3 and: C18:3n–6, reflect their essential source from the diet (2). Despite a large body of literature on longitudinal associations of fatty acid biomarkers with disease risk, most studies have used a single measurement of baseline fatty acids to estimate their prospective associations with disease outcomes. As blood fatty acid levels may change over time, and a single measurement may not reflect long-term status (3–6), it is of interest to understand the variation. However, little is known about the pattern of change in fatty acid composition in a general population over time.

Among prior evidence, in a small study with 50 participants, plasma phospholipid fatty acids were measured from samples obtained around 3 y apart (5). The correlation coefficients ranged between 0.30 and 0.72 for SFAs, between 0.57 and 0.75 for MUFAs, between 0.35 and 0.51 for n–3 PUFAs and between 0.66 and 0.81 for n–6 PUFAs (5). For blood n–3 PUFA, some studies examined the long-term reproducibility of blood fatty acid measurements (3–5), not assessing changes in fatty acid concentrations and their correlates. Trial evidence indicated that both plant-derived n–3 PUFA (C18:3n–3) and marine-derived n–3 PUFAs proportions increased substantially after dietary supplementation with each type of n–3 PUFA over 40 mo (7). Although evaluations of reproducibility and the effects of single fatty acid supplements are important, to our knowledge, no population-based epidemiological study has evaluated the changes in fatty acid composition over time and the correlates of the changes in free-living populations. We aimed to estimate the long-term changes in multiple individual plasma phospholipid fatty acids measured in blood samples collected at 3 time points over more than a decade, and to investigate potential correlates of change in fatty acid composition, in a UK population.

## Methods

### Study population

The current analysis used data from the European Prospective Investigation into Cancer and Nutrition (EPIC)-Norfolk cohort study (8). Briefly, EPIC-Norfolk recruited 25,639 men and women aged 40–79 y through general practice registers in Norfolk, England, and all the participants were invited for a baseline clinic visit between 1993 and 1997 (“health check 1” visit). Participants attended for a further health check ∼3 y later (1998–2000), and again, around 13 y after recruitment (2004–2011) (health checks 2 and 3, respectively). The EPIC-Norfolk study was approved by the Norwich District Ethics Committee, and participants gave written informed consent.

As part of each health check, trained nurses took standardized anthropometric measurements on each participant in light clothing without shoes. Blood samples of the participants were taken and kept frozen at −196 °C until being thawed and measured for fatty acid profiles in 2014. We selected at random 771 participants from the cohort whose blood samples were available at all 3 time points. We excluded participants with baseline self-reported cardiovascular disease (*n* = 14) or cancer (*n* = 35), therefore including 722 participants in the present study (**Supplemental Figure 1**). The mean follow-up time between the first visit (baseline, health check 1) and follow-up visits was 3.4 ± 0.64 y at health check 2, and 12.5 ± 1.86 y at health check 3.

### Diet and lifestyle measurement

Participants completed a health and lifestyle questionnaire at the health checks. Habitual diet was assessed by a 130-item semiquantitative food-frequency questionnaire at health checks 1 and 2 (9). The validity of the dietary measures of the food-frequency questionnaire was assessed against 16-d weighed dietary records, 24-h recalls, and selected nutritional biomarkers in subsamples of EPIC-Norfolk (10–12).

We included 23 major food groups in the present analysis (**Supplemental Table 1**). We disaggregated composite dishes into their constituents and classified them into 1 of the major food groups: total fruit, vegetable, dairy, egg, white fish, fatty fish, red meat, and processed meat, as described previously (9). This approach is consistent with that taken in the UK National Diet and Nutrition Survey (13).

### Measurement of plasma phospholipid fatty acids

Plasma phospholipid fatty acids were measured at the Medical Research Council Elsie Widdowson Laboratory at Cambridge (UK), using the plasma samples stored at baseline at −196 °C, a temperature at which fatty acids remain stable (2). The methods for the fatty acid assay and related quality control were described previously (14). Briefly, the plasma phospholipid fraction was separated by solid-phase extraction, followed by hydrolysis and methylation, after which volatile fatty acid methyl esters were obtained. Separations of different fatty acids were conducted by gas chromatography (J&W HP-88, 30 m length, 0.25 mm internal diameter [Agilent Technologies]) equipped with flame ionization detection (7890 N GC [Agilent Technologies]). We identified 37 different fatty acids by comparing their retention times to those of commercial standards, and each fatty acid was expressed as a percentage of total phospholipid fatty acids (mol percentage). Among 27 individual fatty acids with mean relative concentrations >0.05%, we identified 11 PUFAs, 9 SFAs, 5 MUFAs, and 2 individual trans-fatty acids (TFAs). We further calculated relative concentrations of fatty acid groups by summing individual fatty acids based on prior knowledge about different features of each fatty acid: odd-chain SFAs (C15:0 + C17:0), even-chain SFAs (C14:0 + C16:0 + C18:0), very-long-chain SFAs (C20:0 + C22:0 + C23:0 + C24:0), plant n–3 PUFA (C18:3n–3), marine n–3 PUFAs (C20:5n–3 + C22:5n–3 + C22:6n–3), n–6 PUFAs (C18:2n–6c + C18:3n–6 + C20:2n–6 + C20:3n–6 + C20:4n–6 + C22:4n–6 + C22:5n–6), MUFAs (C16:1 + C17:1 + C18:1n–9c + C20:1n–9 + C24:1), and TFAs (C18:1n–9t + C18:2n–6t).

### Statistical analyses

All analyses were performed using Stata (version 14, StataCorp). Plasma phospholipid fatty acids were winsorized based on 3 SDs, and natural-log-transformed. Intraclass correlation coefficients (ICCs) for fatty acids and fatty acid groups were calculated. Mixed-effects linear regression models were used to estimate the annual changes in fatty acids and fatty acid groups over 13 y from the first to third health check, in which repeated measures of each fatty acid variable were incorporated using random intercepts, with residuals assumed to have an autoregressive correlation structure. Annual relative changes (percentage) in mol percentage of each fatty acid variable were estimated, adjusted for year of recruitment, sex, and baseline variables of age, BMI (in kg/m^2^), physical activity, smoking status, alcohol drinking, education level, social class, total energy intake, fish oil supplements, and intakes of 23 food groups (listed in Supplementary Table 1).

We investigated potential dietary correlates of change in 8 prespecified plasma fatty acid groups (odd-chain SFA, even-chain SFA, very-long-chain SFA, marine n–3 PUFA, plant n–3 PUFA, n–6 PUFA, MUFA, and TFA). As a primary analysis, we estimated the association of change in dietary factors (exposure variable) with simultaneous change in each fatty acid group between the first and second health check (outcome variable). We defined this to be the primary analysis because we considered it to be the most biologically plausible approach, consistent with a previous study evaluating repeated measures of diet, lifestyle, and body weight (15). Mixed-effects linear regression models were used to estimate the annual relative difference (percentage) in change (in mol percentage) of each of the 8 prespecified fatty acid groups, per 1 standard serving/d/y increase in the 23 food groups between 2 health checks. These models were adjusted for year of recruitment, sex, follow-up duration, BMI change between the first 2 health checks, baseline variables of age, BMI, physical activity, smoking status, educational level, alcohol drinking, social class, total energy intake, fish oil supplements intake, corresponding fatty acid group, and food group, and also mutually adjusted for the change in all the other food groups and their interactions with follow-up duration.

Although the primary analysis investigated the association between changes in food consumption and changes in fatty acid concentrations between health checks 1 and 2, we also performed secondary analyses to estimate *1*) associations between baseline food consumption and subsequent changes in each fatty acid group across the 3 health checks (prevalent change model (15)), and *2*) associations of change in dietary factors between health checks 1 and 2 with change in each fatty acid group between health checks 2 and 3 (lagged-change model (15)). We also investigated associations of changes in food group subtypes, including different vegetable types (starchy and nonstarchy) and dairy food intake (low-fat dairy, high-fat dairy, milk, yogurt, cheese, other dairy, butter) with change in each fatty acid group between health checks 1 and 2 (i.e., an extension of the primary analysis). Finally, we estimated association of change in BMI with change in each plasma phospholipid fatty acid groups, and prospective associations of baseline age, BMI, smoking status, and physical activity with change in each plasma fatty acid group.

## Results

### Population characteristics

**Table 1** shows the baseline characteristics of the study participants, who had a mean age of 55.5 ± 8.1 y, and a mean BMI of 25.5 ± 3.6 kg/m^2^. The distributions of food group consumption and plasma phospholipid fatty acids at each health check are summarized in **Supplemental Table 2** and **Table 2**. The median marine n–3 PUFA concentrations were 6.28 mol percentage and 7.45 mol percentage at health checks 1 and 3, respectively; and n–6 PUFA, 35.6% and 34.5 mol percentage, respectively (Table 2). At health check 1, 33% of participants took fish oil supplements, with 27.1% among participants aged <55 y and 38.8% among those aged ≥55y. ICCs of the fatty acids estimated from the 3 health checks were low to moderate, lowest for C17:1 (ICC = 0.09), and highest for C20:4n–6 + C20:3n–3 (ICC = 0.60) (**Supplemental Table 3**).

**TABLE 1:** Population characteristics of the participants at baseline (health check 1) of the European Prospective Investigation into Cancer and Nutrition-Norfolk study (*n* = 722)

	Mean ± SD or percentage
Age^1^, y	55.5 ± 8.1
BMI^1^, kg/m^2^	25.5 ± 3.6
Sex, % men	39.1
Alcohol intake^1^, units/wk	6.7 ± 7.04
Total energy intake^1^, kJ/d	8492 ± 2381
Physical activity, %	
Inactive	20.9
Moderately inactive	31.2
Moderately active	25.6
Active	22.3
Education level, %	
No reported qualifications	24.6
O level or equivalent (to age 16 y)	13.0
A level or equivalent (to age 18 y)	43.4
Degree or higher	19.0
Social class, %	
Professional	8.6
Managerial and technical	41.2
Skilled nonmanual	18.3
Skilled manual	19.8
Semiskilled	10.0
Unskilled	2.09
Smoking status, %	
Never	57.6
Former	36.9
Current	5.5
Alcohol drinking status, %	
Never	1.7
Former	4.7
Current	93.6
Fish oil supplement use, %	33.0

^1^These variables are presented as means ± SDs; others are presented as percentages.

**TABLE 2:** Distribution of plasma phospholipid fatty acids (mol%) across the 3 time points from 1993–1997 to 2004–2011 in European Prospective Investigation into Cancer and Nutrition-Norfolk^1^

Types of fatty acids	Health check 1 (1993–1997)^2^	Health check 2 (1998–2000)^2^	Health check 3 (2004–2011)^2^	Change between health check 1 and 2	Change between health check 2 and 3
Odd-chain SFA	0.65 (0.58, 0.72)	0.65 (0.56, 0.75)	0.60 (0.54, 0.67)	0.01 (−0.07, 0.09)	−0.05 (−0.14, 0.03)
C15:0	0.23 (0.19, 0.26)	0.21 (0.18, 0.25)	0.20 (0.18, 0.24)	−0.01 (−0.04, 0.02)	−0.01 (−0.04, 0.02)
C17:0	0.42 (0.37, 0.47)	0.42 (0.37, 0.51)	0.40 (0.35, 0.44)	0 (−0.04, 0.08)	−0.03 (−0.1, 0.02)
Even-chain SFA	45.3 (44.6, 46.0)	45.1 (44.5, 45.7)	45.0 (44.4, 45.5)	−0.15 (−0.85, 0.70)	−0.19 (−0.89, 0.47)
C14:0	0.39 (0.33, 0.45)	0.40 (0.34, 0.46)	0.39 (0.33, 0.45)	0 (−0.06, 0.08)	−0.01 (−0.08, 0.06)
C16:0	30.5 (29.6, 31.4)	30.5 (29.7, 31.5)	30.4 (29.7, 31.2)	0.18 (−0.70, 0.99)	−0.09 (−0.99, 0.74)
C18:0	14.4 (13.6, 15.1)	14.2 (13.4, 14.8)	14.1 (13.4, 14.8)	−0.24 (−0.91, 0.41)	−0.04 (−0.78, 0.79)
Very-long-chain SFA	0.71 (0.64, 0.80)	0.67 (0.60, 0.72)	0.67 (0.61, 0.77)	−0.04 (−0.13, 0.03)	0.01 (−0.07, 0.11)
C20:0	0.13 (0.12, 0.15)	0.12 (0.11, 0.14)	0.12 (0.11, 0.13)	−0.01 (−0.03, 0.01)	−0.01 (−0.02, 0.01)
C22:0	0.25 (0.22, 0.28)	0.21 (0.19, 0.24)	0.21 (0.19, 0.24)	−0.03 (−0.07, 0)	0 (−0.03, 0.04)
C23:0	0.11 (0.09, 0.13)	0.10 (0.09, 0.11)	0.11 (0.10, 0.13)	−0.01 (−0.03, 0.01)	0.01 (0, 0.03)
C24:0	0.22 (0.20, 0.25)	0.22 (0.20, 0.24)	0.23 (0.20, 0.27)	0 (−0.03, 0.02)	0.01 (−0.02, 0.05)
Plant n–3 PUFA (C18:3n–3)	0.31 (0.23, 0.39)	0.34 (0.27, 0.42)	0.30 (0.25, 0.37)	0.03 (−0.07, 0.13)	−0.03 (−0.13, 0.06)
Marine n–3 PUFA	6.28 (5.21, 7.46)	6.57 (5.47, 7.84)	7.45 (6.15, 8.80)	0.27 (−0.74, 1.38)	0.72 (−0.5, 1.86)
C20:5n–3	1.16 (0.87, 1.57)	1.33 (1.00, 1.74)	1.65 (1.27, 2.34)	0.15 (−0.20, 0.54)	0.26 (−0.12, 0.86)
C22:5n–3	0.99 (0.85, 1.12)	1.05 (0.93, 1.20)	1.10 (0.97, 1.24)	0.07 (−0.05, 0.20)	0.05 (−0.09, 0.20)
C22:6n–3	4.08 (3.37, 4.88)	4.16 (3.34, 5.03)	4.54 (3.74, 5.39)	0.03 (−0.60, 0.64)	0.34 (−0.46, 1.08)
n–6 PUFA	35.6 (33.8, 37.2)	35.2 (33.4, 36.7)	34.5 (32.7, 36.1)	−0.43 (−2.18, 1.25)	−0.63 (−2.37, 1.14)
C18:2n–6c	23.3 (21.3, 25.3)	22.5 (20.6, 24.6)	21.4 (19.3, 23.2)	−0.83 (−2.72, 1.26)	−1.23 (−3.16, 0.89)
C18:3n–6	0.08 (0.06, 0.11)	0.08 (0.06, 0.11)	0.09 (0.07, 0.12)	0 (−0.02, 0.03)	0.01 (−0.02, 0.04)
C20:2n–6	0.37 (0.33, 0.42)	0.37 (0.34, 0.41)	0.34 (0.31, 0.38)	0 (−0.04, 0.04)	−0.03 (−0.07, 0)
C20:3n–6	2.94 (2.51, 3.45)	3.04 (2.60, 3.57)	3.12 (2.61, 3.61)	0.12 (−0.29, 0.49)	0.08 (−0.40, 0.44)
C20:4n–6 + C20:3n–3	8.17 (7.22, 9.25)	8.52 (7.37, 9.58)	8.77 (7.69, 10.3)	0.19 (−0.55, 1.10)	0.46 (−0.59, 1.49)
C22:4n–6	0.25 (0.21, 0.29)	0.25 (0.22, 0.30)	0.24 (0.20, 0.28)	0.01 (−0.03, 0.04)	−0.01 (−0.05, 0.02)
C22:5n–6	0.14 (0.12, 0.18)	0.15 (0.12, 0.18)	0.14 (0.11, 0.18)	0.01 (−0.02, 0.03)	0 (−0.03, 0.03)
MUFA	10.3 (9.40, 11.4)	10.8 (9.90, 11.7)	10.9 (10.1, 12.0)	0.45 (−0.60, 1.36)	0.23 (−0.75, 1.32)
C16:1	0.49 (0.39, 0.62)	0.51 (0.42, 0.64)	0.56 (0.45, 0.68)	0.03 (−0.06, 0.12)	0.04 (−0.07, 0.14)
C17:1	0.06 (0.04, 0.07)	0.05 (0.03, 0.06)	0.05 (0.04, 0.06)	−0.01 (−0.03, 0.01)	0 (−0.01, 0.02)
C18:1n–9c	9.08 (8.29, 10.1)	9.61 (8.66, 10.5)	9.68 (8.89, 10.6)	0.38 (−0.50, 1.26)	0.17 (−0.78, 1.07)
C20:1n–9	0.29 (0.25, 0.34)	0.26 (0.21, 0.31)	0.23 (0.20, 0.27)	−0.03 (−0.09, 0.03)	−0.03 (−0.08, 0.02)
C24:1	0.32 (0.29, 0.38)	0.36 (0.31, 0.40)	0.41 (0.35, 0.48)	0.03 (−0.03, 0.08)	0.05 (−0.01, 0.12)
Trans-fatty acids	0.39 (0.30, 0.52)	0.33 (0.26, 0.43)	0.14 (0.12, 0.17)	−0.05 (−0.16, 0.05)	−0.19 (−0.28, −0.11)
C18:1n–9t	0.32 (0.24, 0.45)	0.27 (0.20, 0.37)	0.08 (0.07, 0.11)	−0.04 (−0.15, 0.05)	−0.18 (−0.27, −0.1)
C18:2n–6t	0.07 (0.06, 0.08)	0.06 (0.05, 0.07)	0.05 (0.04, 0.06)	−0.01 (−0.02, 0)	−0.01 (−0.02, 0)

^1^Data are presented as median (interquartile range). The units of all the fatty acids or fatty acid groups are mol%. The sample size used in this table was 669 after excluding any missing data at any of the health check.

^2^Health check 1 represents the time period of the baseline recruitment (1993–1997); these participants were followed up 3–4 y after recruitment (health check 2, 1998–2000), and on average 13 y after recruitment (health check 3, 2004–2011).

### Change in plasma phospholipid fatty acids, estimated using measures from 3 time points

**FIGURE 1:**
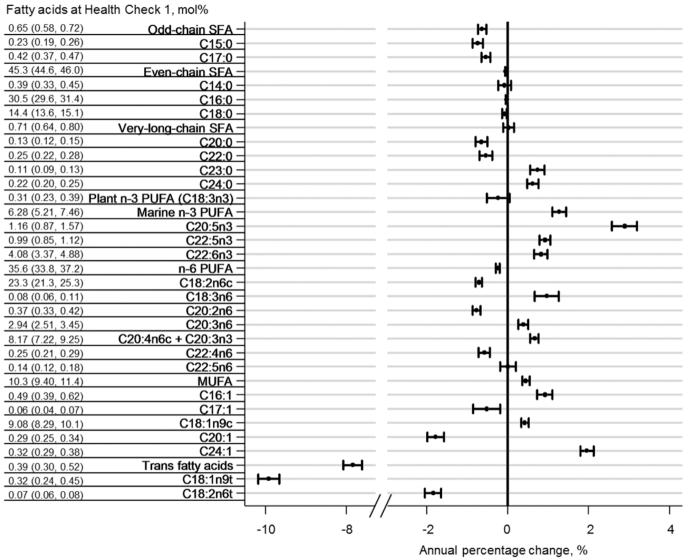
Annual percentage change in 27 plasma phospholipid fatty acids and fatty acid groups in the EPIC-Norfolk study over 13 y. Error bars represent 95% CIs. In the mixed-effects linear regression models, percentage changes in each fatty acid or fatty acid group were adjusted for year of recruitment, sex, and baseline variables of age, BMI, physical activity, smoking status, alcohol drinking, educational level, social class, total energy intake, fish oil supplements, and intakes of 23 food groups (fruit, vegetable, legume, total dairy, eggs, white fish, fatty fish, red meat, white meat, processed meat, liver potatoes, cereal, bread, sweets, nuts and seeds, tea, coffee, fruit juice, sugar-sweetened beverage, alcohol, margarine, and vegetable oil). The column on the left lists the baseline levels of fatty acids and fatty acid groups.

There was a relative decrease in TFAs over time (−7.84%/y; 95% CI: −8.07%, −7.61%) (**Figure 1**), which corresponded to a decrease from 0.39 mol percentage at baseline to 0.14 mol percentage at health check 3 (Table 2). There were also relative decreases in levels of odd-chain SFAs (−0.63%/y; 95% CI: −0.73%, −0.52%), even-chain SFAs (−0.05%/y; 95% CI: −0.06%, −0.03%), and n–6 PUFAs (−0.25%/y; 95% CI: −0.29%, −0.20%). In contrast, there were relative increases in marine n–3 PUFAs (+1.28%/y; 95% CI: 1.11%, 1.44%), and MUFAs (+0.45%/y; 95% CI: 0.37%, 0.54%), but no changes in very-long-chain SFAs or plant n–3 PUFA (Figure 1).

The relative changes in individual fatty acids varied substantially within some fatty acid groups. For example, among very-long-chain SFAs, both C20:0 and C22:0 decreased (−0.65% [95% CI: −0.79%, −0.50%] and −0.54% [95% CI: −0.69%, −0.38%], respectively, per year), whereas both C23:0 and C24:0 increased (+ 0.74% [95% CI: 0.56%, 0.92%] and + 0.62% [95% CI: 0.49%, 0.76%], respectively). A similar variation was seen among the individual fatty acids of the n–6 PUFA and MUFA groups (Table 2, Figure 1).

### Dietary factors and change in plasma phospholipid fatty acid groups

**FIGURE 2:**
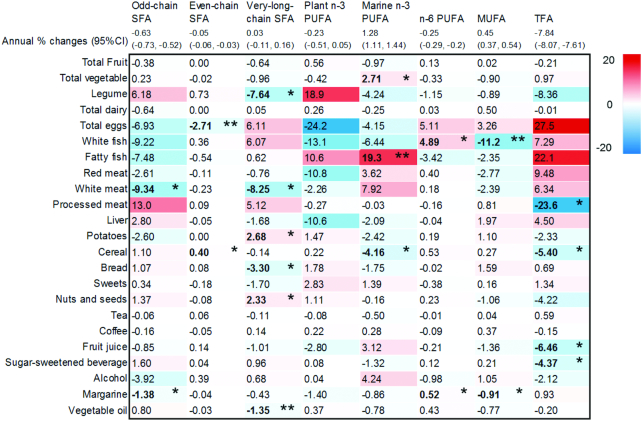
Associations between changes in food groups and changes in plasma fatty acid groups from 1993–1997 to 1998–2000 (between study health checks 1 and 2): European Prospective Investigation into Cancer and Nutrition-Norfolk Study. Mixed-effects linear regression models were used in the analyses. Each value represents a mean relative difference (%) in change/y in mol% of each fatty acid group, per 1-standard serving/d/y increase in food groups (*P < 0.05 and **P < 0.01). Red and blue boxes indicate positive and negative associations, respectively, of change/y in food consumption with change/y in mol% of fatty acids. Mean annual changes in mol% of each fatty acid group are presented in the top row (above the box). Serving sizes were defined as 10 g/d for margarine, liver, nuts, and seeds; as 1 g/d for vegetable oil and 10 units/wk for alcohol; and as 100 g/d for the other food groups. All the estimates were mutually adjusted for changes in food groups and baseline consumption levels of those foods, and adjusted for baseline levels of the given fatty acid and other potential confounders (see Methods text for detail). TFA, trans-fatty acids.

**Figure 2** (and **Supplemental Table 4**) shows the changes in fatty acid levels by the intakes of different food groups. For example, a mean increase in fatty fish intake of 100 g/d was associated with a 19.3% (95% CI: 4.43%, 36.3%) greater annual increase in marine n–3 PUFAs (Figure 2), and with a greater annual increase in each of C20:5n–3 and C22:6n–3 (**Supplemental Figure 2**). The mean change in marine n–3 PUFAs was +1.28 mol percentage, and this increase was greater among those who increased fish consumption over time. An increase in margarine intake by 10 g/d and an increase in white fish by 100 g/d were associated with smaller decreases in plasma n–6 PUFAs: 0.52% (0.12%, 0.93%) and 4.89% (0.13%, 9.86%), respectively. White fish was only associated with C18:2n–6, and not with the other individual n–6 PUFAs, whereas margarine was not associated with any individual n–6 PUFAs (Supplemental Figure 2). In addition, an increase in intakes of some food groups was associated with decreases in TFAs. Per 100 g/d higher intake, there were larger reductions in TFA as follows: TFA –23.6% (−41.2%, −0.77%) with processed meat, −5.40% (−10.3%, −0.22) with cereals, −6.46% (−12.1%, −0.45%) with fruit juice, and −4.37% (−7.80%, −0.82%) with sugar-sweetened beverages (Figure 2, Supplemental Table 4).

Increases in potato and nut and seed intakes were positively associated with total very-long-chain SFAs, by 2.68% per 100 g/d (95% CI: 0.08%, 5.35%) and 2.33% (0.15%, 4.55%), respectively.

In secondary prospective analyses relating baseline dietary consumption to subsequent changes in fatty acid concentrations (prevalent change model, **Supplemental Figure 3**, **Supplemental Table 5**), a 100 g/d higher baseline intake of sweets was associated with a greater annual decrease in TFAs (−0.96%; 95% CI: −1.32%, −0.59%). The lagged-change model suggested that a 100 g/d higher intake of sugar-sweetened beverages between health check 1 and 2 was associated with an attenuated annual decrease in even-chain SFAs between health check 2 and 3 (0.14%; 95% CI: 0.06%, 0.22%) (**Supplemental Figure 4**, Supplemental Table 5).

Results for food group subtypes (dairy and vegetable) are presented in **Supplemental Figure 5**. Increases in cheese and starchy vegetable intakes were associated with a greater increase in very-long-chain SFAs. Dairy intakes except for low-fat dairy and milk were associated with smaller decreases in odd-chain SFAs.

### Age, BMI, and change in plasma phospholipid fatty acid groups

A higher baseline age was associated with lower even-chain SFAs and very-long-chain SFAs, and with a lower decrease in n–6 PUFAs and TFAs (**Supplemental Table 6**). Per 1 kg/m^2^ increase in BMI over time was associated with 0.39% (0.01%, 0.78%) higher levels in even-chain SFA group per year, but change in physical activity was not associated with any changes in fatty acids (**Supplemental Figure 6**).

## Discussion

With a unique data set of repeated measures of plasma phospholipid fatty acids over a 13-year period, we identified patterns in the change in different fatty acid groups: relative concentrations of odd-chain SFAs, even-chain SFAs, n–6 PUFAs, and TFAs decreased over time, whereas relative concentrations of marine n–3 PUFAs and MUFAs increased over time, and no change was observed for very-long-chain SFAs or plant n–3 PUFAs. These changes were independent of baseline diet, lifestyle factors, adiposity, and other potential confounders.

Blood marine n–3 PUFAs increased over time, consistent with increases in population-level dietary intakes of marine n–3 PUFAs reported in the UK national survey (16, 17), and also with an increase in the use of fish oil supplements in the present study. In addition, age may play a potential role in the change in marine n–3 PUFAs over time, given that the aging process may affect both the synthesis and metabolism of marine n–3 PUFAs (18, 19). There was evidence suggesting that aging may change marine n–3 PUFA homeostasis, and elderly people tended to have higher levels of marine n–3 PUFAs, independent of dietary intake (18, 19). Taken together, the above findings highlighted the important role of both diet and aging in the change in marine n–3 PUFA over time. The association between change in fatty fish intake and change in blood marine n–3 PUFAs is biologically plausible, because plasma phospholipid marine n–3 PUFAs are robust biomarkers of the dietary fish/fish oil intake (2). In addition, the finding for marine n–3 PUFAs confirmed the validity of the primary analysis.

TFAs exhibited the most substantial change (−7.84%/y) over time. Plasma C18:1n–9t and C18:2n–6t are likely to originate from foods made with partially hydrogenated vegetable oils (including biscuits, chips and popcorn, fried foods, and bakery foods) (20–22), with a small proportion contributed by ruminant sources (20, 21, 23). We found that a higher intake of a variety of dietary factors (processed meat, cereals, fruit juice, sugar-sweetened beverages, and cheese) during follow-up and a higher intake of baseline sweets contributed to the decrease in TFAs over time. These associations may not reflect biology, and we speculate that these food groups or related dietary patterns increased among individuals who were more likely to reduce their trans-fat intake as a result of known public health efforts to reduce trans fat in processed foods (24). Over the past 2 decades, recognizing their adverse health effects, trans fats have been gradually removed from food products by food manufacturers in many countries including the UK, and this time period aligns well with the timing of the 3 health checks when the blood samples were collected in the present study (24, 25). Meanwhile, high-fat dairy intake, a source of ruminant TFAs, also decreased gradually in the UK (26). Moreover, dietary intakes of TFAs have been observed to decline over time (16, 17, 27). Therefore, the decrease in TFA biomarkers in this study was likely to reflect decreases in TFA contents in a variety of foods and in dietary TFA intake over years.

Similar to TFAs, we also observed decreases in odd-chain and even-chain SFAs over time. Although even-chain SFAs (major SFAs in circulation and diet) reflect not only dietary SFAs, but also endogenous synthesis (28), these results corroborated a decline in dietary SFAs in the UK between 1990 and 2010, as reported in a global survey (27), and in the UK National Diet and Nutrition Survey (16, 17).

We did not find any evidence of change in very-long-chain SFAs over time. Very-long-chain SFAs originate from endogenous biological synthesis (29), whereas their dietary sources remain unclear. The present study indicated that potatoes, nuts and seeds, cheese, and starchy vegetables might be possible dietary correlates of plasma phospholipid very-long-chain SFA levels.

We found that baseline age was related to changes in various fatty acid groups over time. Older individuals may be more health-conscious and more likely to be motivated to adhere to dietary guidelines to decrease their dietary SFA intake and increase overall PUFA intake, which may explain the findings between baseline age and change in SFAs and n–6 PUFAs. The current finding that change in BMI was positively associated with change in even-chain SFAs was consistent with prior evidence that a higher BMI might promote de novo lipogenesis (30), a mechanism for endogenous synthesis of even-chain SFAs in the liver.

As suggested by the present findings, a longitudinal analysis linking baseline fatty acid distribution to future disease risk should be interpreted with caution. For example, participants with a low blood n–3 PUFA level at baseline may be those with a low fish intake and not taking fish oil supplements. As these individuals get older and become more health-conscious, they may increase dietary fish or supplement intake and experience increasing blood marine n–3 PUFA levels over time. Therefore, it could be hypothesized that a true effect of low n–3 PUFA on disease incidence would be underestimated under the assumption that the number of individuals with truly low n–3 PUFA would decrease over time.

There were several limitations of the present study. Because we measured the plasma phospholipid fraction with long-term stored samples, degradation of some fatty acid compositions may be of concern. Although blood samples in EPIC-Norfolk were stored in liquid nitrogen at low temperature (−196 °C), and fatty acids were postulated to be stable during storage, direct evidence supporting the storage stability of blood fatty acids at such a low temperature over 10 y was lacking (2). We noted that 1 prior small study among 22 participants suggested that storage at −80 °C up to 10 y did not substantially influence serum fatty acid profiles (31). The analyses were based on relative concentrations of plasma phospholipid fatty acids, which were dependent on each other, and could not distinguish changes in absolute levels. However, results based on relative concentrations have been widely used, making these results comparable with the majority of published studies. Finally, although we mutually adjusted for potential correlates of changes in fatty acids, the potential for bias due to measurement error and residual confounding cannot be excluded. The strengths of the present study included repeated measures of 27 plasma fatty acid data over a long period of time (mean 13 y) in a sample of free-living individuals, representative of the general UK population. In addition, with repeated measures of dietary data including 23 food groups, we were able to assess change in diet and fatty acid compositions simultaneously, and identify potential dietary correlates of plasma fatty acids over a long period.

In conclusion, these results suggest that changes in fatty acid concentrations over time are likely to be influenced by a combination of biological factors such as aging and dietary factors, particularly for the fatty acids that are affected by current dietary advice. The results about the direction of the changes in individual fatty acids and fatty acid groups over time in a general population could help to guide the interpretation of findings from other observational studies with a single measurement of fatty acid composition at baseline. A further investigation into changes in circulating fatty acids during follow-up and their associations with future disease risk would improve understanding of roles of fatty acids in health and disease incidence.

## Supplementary Material

nqz030_Supplemental_File

## Notes

Medical Research Council Epidemiology Unit MC_UU_12015/1 and MC_UU_12015/5; Medical Research Council Human Nutrition Research MC_UP_A090_1006; Cambridge Lipidomics Biomarker Research Initiative G0800783; NIHR Biomedical Research Centre Cambridge: Nutrition, Diet, and Lifestyle Research Theme (IS-BRC-1215-20014). Dr Ju-Sheng Zheng has received funding from the European Union's Horizon 2020 research and innovation program under the Marie Sklodowska-Curie grant agreement No. 701708.

Supplemental Tables 1–6 and Supplemental Figures 1–6 are available from the “Supplementary data” link in the online posting of the article and from the same link in the online table of contents at https://academic.oup.com/ajcn/.

Abbreviations used: EPIC, European Prospective Investigation into Cancer and Nutrition; ICC, intraclass correlation coefficient; TFA, trans-fatty acid.

## References

[1] Fats and fatty acids in human nutrition. Report of an expert consultation. FAO Food Nutr Pap. 2010;91:1–166. 21812367

[2] Hodson L, Skeaff CM, Fielding BA Fatty acid composition of adipose tissue and blood in humans and its use as a biomarker of dietary intake. Prog Lipid Res. 2008;47:348–80. 18435934 10.1016/j.plipres.2008.03.003

[3] Mozaffarian D, Lemaitre RN, King IB, Song X, Spiegelman D, Sacks FM, Rimm EB, Siscovick DS Circulating long-chain omega-3 fatty acids and incidence of congestive heart failure in older adults: The cardiovascular health study: A cohort study. Ann Intern Med. 2011;155:160–70. 21810709 10.1059/0003-4819-155-3-201108020-00006 PMC3371768

[4] Djousse L, Petrone AB, Weir NL, Hanson NQ, Glynn RJ, Tsai MY, Gaziano JM Repeated versus single measurement of plasma omega-3 fatty acids and risk of heart failure. Eur J Nutr. 2014;53:1403–8. 24395612 10.1007/s00394-013-0642-3 PMC4085145

[5] Ma J, Folsom AR, Eckfeldt JH, Lewis L, Chambless LE Short- and long-term repeatability of fatty acid composition of human plasma phospholipids and cholesterol esters. The Atherosclerosis Risk in Communities (ARIC) Study Investigators. Am J Clin Nutr. 1995;62:572–8. 7661119 10.1093/ajcn/62.3.572

[6] Zeleniuch-Jacquotte A, Chajès V, Van Kappel AL, Riboli E, Toniolo P Reliability of fatty acid composition in human serum phospholipids. Eur J Clin Nutr. 2000;54:367–72. 10822282 10.1038/sj.ejcn.1600964

[7] Kromhout D, Giltay EJ, Geleijnse JM n–3 Fatty acids and cardiovascular events after myocardial infarction. N Engl J Med. 2010;363:2015–26. 20929341 10.1056/NEJMoa1003603

[8] Day N, Oakes S, Luben R, Khaw KT, Bingham S, Welch A, Wareham N EPIC-Norfolk: Study design and characteristics of the cohort. European Prospective Investigation of Cancer. Br J Cancer. 1999;80 Suppl 1:95–103. 10466767

[9] Mulligan AA, Luben RN, Bhaniani A, Parry-Smith DJ, O'Connor L, Khawaja AP, Forouhi NG, Khaw KT A new tool for converting food frequency questionnaire data into nutrient and food group values: FETA research methods and availability. BMJ Open. 2014; 10.1136/bmjopen-2013-004503 PMC3975761 24674997

[10] Bingham SA, Cassidy A, Cole TJ, Welch A, Runswick SA, Black AE, Thurnham D, Bates C, Khaw KT, Key TJ Validation of weighed records and other methods of dietary assessment using the 24 h urine nitrogen technique and other biological markers. Br J Nutr. 1995;73:531–50. 7794870 10.1079/bjn19950057

[11] Bingham S, Welch A, Day K, Cassidy A Comparison of dietary assessment methods in nutritional epidemiology: Weighed records v. 24 h recalls, food-frequency questionnaires and estimated-diet records. Br J Nutr. 1994;72:619–43. 7986792 10.1079/bjn19940064

[12] Bingham SA, Gill C, Welch A, Cassidy A, Runswick SA, Oakes S, Lubin R, Thurnham DI, Key TJ, Roe L et al. Validation of dietary assessment methods in the UK arm of EPIC using weighed records, and 24-hour urinary nitrogen and potassium and serum vitamin C and carotenoids as biomarkers. Int J Epidemiol. 1997;26 Suppl 1:S137–51. 9126542 10.1093/ije/26.suppl_1.s137

[13] Fitt E, Mak TN, Stephen AM, Prynne C, Roberts C, Swan G, Farron-Wilson M Disaggregating composite food codes in the UK National Diet and Nutrition Survey food composition databank. Eur J Clin Nutr. 2010;64:S32–6. 21045846 10.1038/ejcn.2010.207

[14] Wang LY, Summerhill K, Rodriguez-Canas C, Mather I, Patel P, Eiden M, Young S, Forouhi NG, Koulman A Development and validation of a robust automated analysis of plasma phospholipid fatty acids for metabolic phenotyping of large epidemiological studies. Genome Med. 2013;5:39. 23618465 10.1186/gm443 PMC3706814

[15] Smith JD, Hou T, Hu FB, Rimm EB, Spiegelman D, Willett WC, Mozaffarian D A comparison of different methods for evaluating diet, physical activity, and long-term weight gain in 3 prospective cohort studies. J Nutr. 2015;145(11):2527–34. 26377763 10.3945/jn.115.214171 PMC4620721

[16] Pot GK, Prynne CJ, Roberts C, Olson A, Nicholson SK, Whitton C, Teucher B, Bates B, Henderson H, Pigott S et al. National Diet and Nutrition Survey: Fat and fatty acid intake from the first year of the rolling programme and comparison with previous surveys. Br J Nutr. 2012;107:405–15. 21767448 10.1017/S0007114511002911 PMC3428836

[17] Henderson L, Gregory J, Irving K, Swan G The national diet and nutrition survey: Adults aged 19–64 years. Vol. 2: Energy, Protein, Carbohydrate, Fat and Alcohol Intake. London: The Stationery Office; 2003.

[18] Chouinard-Watkins R, Brenna JT, Meyer BJ, Cunnane SC Does aging change docosahexaenoic acid homeostasis? Implications for the challenge to cognitive health in the elderly DHA and aging-related cognitive decline. OCL. 2011;18:175–80.

[19] De Groot RHM, Van Boxtel MPJ, Schiepers OJG, Hornstra G, Jolles J Age dependence of plasma phospholipid fatty acid levels: Potential role of linoleic acid in the age-associated increase in docosahexaenoic acid and eicosapentaenoic acid concentrations. Br J Nutr. 2009;102:1058–64. 19402937 10.1017/S0007114509359103

[20] Dhaka V, Gulia N, Ahlawat KS, Khatkar BS Trans fats-sources, health risks and alternative approach—A review. J Food Sci Technol. 2011;48:534–41. 23572785 10.1007/s13197-010-0225-8 PMC3551118

[21] Thiébaut ACM, Rotival M, Gauthier E, Lenoir GM, Boutron-Ruault M-C, Joulin V, Clavel-Chapelon F, Chajès V Correlation between serum phospholipid fatty acids and dietary intakes assessed a few years earlier. Nutr Cancer. 2009;61:500–9. 19838922 10.1080/01635580802710717

[22] Micha R, King IB, Lemaitre RN, Rimm EB, Sacks F, Song X, Siscovick DS, Mozaffarian D Food sources of individual plasma phospholipid trans fatty acid isomers: The Cardiovascular Health Study. Am J Clin Nutr. 2010;91:883–93. 20219966 10.3945/ajcn.2009.28877 PMC2844676

[23] Stender S, Astrup A, Dyerberg J Ruminant and industrially produced trans fatty acids: Health aspects. Food Nutr Res. 2008;52, 10.3402/fnr.v52i0.1651. PMC2596737 19109659

[24] Roe M, Pinchen H, Church S, Elahi S, Walker M, Farron-Wilson M, Buttriss J, Finglas P Trans fatty acids in a range of UK processed foods. Food Chem. 2013;140:427–31. 23601386 10.1016/j.foodchem.2012.08.067

[25] Downs SM, Thow AM, Leeder SR The effectiveness of policies for reducing dietary trans fat: A systematic review of the evidence. Bull World Health Organ. 2013;91:262–269H. 23599549 10.2471/BLT.12.111468 PMC3629452

[26] Whitton C, Nicholson SK, Roberts C, Prynne CJ, Pot GK, Olson A, Fitt E, Cole D, Teucher B, Bates B et al. National Diet and Nutrition Survey: UK food consumption and nutrient intakes from the first year of the rolling programme and comparisons with previous surveys. Br J Nutr. 2011;106:1899–914. 21736781 10.1017/S0007114511002340 PMC3328127

[27] Micha R, Khatibzadeh S, Shi P, Fahimi S, Lim S, Andrews KG, Engell RE, Powles J, Ezzati M, Mozaffarian D Global, regional, and national consumption levels of dietary fats and oils in 1990 and 2010: A systematic analysis including 266 country-specific nutrition surveys. BMJ. 2014;348:g2272. 24736206 10.1136/bmj.g2272 PMC3987052

[28] Ma W, Wu JHY, Wang Q, Lemaitre RN, Mukamal KJ, Djoussé L, King IB, Song X, Biggs ML, Delaney JA et al. Prospective association of fatty acids in the de novo lipogenesis pathway with risk of type 2 diabetes: The Cardiovascular Health Study. Am J Clin Nutr. 2015;101:153–63. 25527759 10.3945/ajcn.114.092601 PMC4266885

[29] Reddy JK, Hashimoto T Peroxisomal beta-oxidation and peroxisome proliferator-activated receptor alpha: An adaptive metabolic system. Annu Rev Nutr. 2001;21:193–230. 11375435 10.1146/annurev.nutr.21.1.193

[30] Alves MG, Moreira Â, Guimarães M, Nora M, Sousa M, Oliveira PF, Monteiro MP Body mass index is associated with region-dependent metabolic reprogramming of adipose tissue. BBA Clin. 2017;8:1–6. 28567337 10.1016/j.bbacli.2017.05.001 PMC5440253

[31] Matthan NR, Ip B, Resteghini N, Ausman LM, Lichtenstein AH Long-term fatty acid stability in human serum cholesteryl ester, triglyceride, and phospholipid fractions. J Lipid Res. 2010;51:2826–32. 20448292 10.1194/jlr.D007534 PMC2918465

