# Supplementary material for: Changes in plasma phospholipid fatty acid profiles over 13 years and correlates of change: European Prospective Investigation into Cancer and Nutrition-Norfolk Study
Source: Am J Clin Nutr. 2019 Apr 17;109(6):1527–34. doi: 10.1093/ajcn/nqz030 (PMC6537938; doi:10.1093/ajcn/nqz030)

"Changes in plasma phospholipid fatty acids over 13 years and correlates of change: EPIC-Norfolk Study"

Authors: Ju-Sheng Zheng, Fumiaki Imamura, Stephen J Sharp, Albert Koulman, Jules Griffin, Angela A Mulligan, Robert Luben, Kay-Tee Khaw, Nicholas J Wareham, Nita G Forouhi

Online Supplemental Tables

Supplemental Table 1 Food item components of food groups

Supplemental Table 2 Distribution and change of food group consumption from 1993-1997 to 1998-2000 in the EPIC-Norfolk study

Supplemental Table 3 Intraclass correlation coefficient for the repeated measure of plasma phospholipid fatty acids across the three time points from 1993-1997 to 2004-2011

Supplemental Table 4 Association of changes in food groups with changes in plasma phospholipid fatty acid groups from 1993-1997 to 1998-2000

Supplemental Table 5 Association of changes in food groups with changes in plasma phospholipid fatty acid groups in the prevalent change and lagged-change models

Supplemental Table 6 Association of baseline age, body mass index, smoking status and physical activity with annual percentage change of plasma phospholipid fatty acid groups

Online Supplemental Figures

Supplemental Figure 1 Flow-chart of the present study.

Supplemental Figure 2 Associations between changes in food groups and changes in individual plasma fatty acids from 1993-1997 to 1998-2000. Mixed-effects linear regression models were used in the analyses. Values represent an average relative difference (%) in changes/year in mol% of each individual saturated fatty acids (A), polyunsaturated fatty acids (B), monounsaturated fatty acids and trans fatty acids (C) per one serving/day/year increase in food groups (* $p<0.05$ and ** $p<0.01$). Serving sizes were defined as 10 g/d for margarine, liver, nuts and seeds; as 1 g/d for vegetable oil and 10 units/wk for alcohol; and 100 g/day for the other food groups. Red and blue boxes indicate positive and negative associations, respectively, of changes/year in food consumption with changes/year in mol% of fatty acids. Average annual changes in mol% of each fatty acid group are presented on the top. All the estimates were mutually adjusted for changes in food groups and baseline consumption levels of those and adjusted for baseline levels of a fatty acid and other potential confounders (see text in details).

Supplemental Figure 3 Associations of baseline 23 food groups with annual percentage change in each plasma fatty acid group from 1993-1997 to 2004-2011. Mixed-effects linear regression models were used in the analyses. Each value represents an average relative difference (%) in changes/year in mol% of each fatty acid group from 1993-1997 to 2004-2011, per 1-standard serving/day higher in baseline food groups, with mutual adjustment for the baseline food groups, fatty acid change over the same time period and other potential confounders (* $p<0.05$ and ** $p<0.01$). Serving sizes were defined as 10g/d for margarine, liver, nuts and seeds; 1g/d for vegetable oil; 10 units/wk for alcohol and 100g/d for other food groups. Values in blue font represent negative

associations; values in red font represent positive associations. SFA, saturated fatty acids; PUFA, polyunsaturated fatty acids; MFUA, monounsaturated fatty acids; TFA, trans fatty acids.

Supplemental Figure 4 Association between changes in 23 food groups from 1993-1997 to 1998-2000 and subsequent changes in each plasma fatty acid group from 1998-2000 to 2004-2011. Mixed-effects linear regression models were used in the analyses. Each value represents an average difference (%) in changes/year in mol% of each fatty acid group between 1998-2000 and 2004-2011, per annual 1-standard serving/day increase in each food group between 1993-1997 and 1998-2000, with mutual adjustment for changes in food groups and baseline consumption levels of those and adjusted for baseline levels of a fatty acid and other potential confounders (* p<0.05 and ** p<0.01). Serving sizes were defined as 10g/d for margarine, liver, nuts and seeds; 1g/d for vegetable oil; 10 units/wk for alcohol and 100g/d for other food groups. Values in blue font represent negative associations; values in red font represent positive associations. SFA, saturated fatty acids; PUFA, polyunsaturated fatty acids; MFUA, monounsaturated fatty acids; TFA, trans fatty acids.

Supplemental Figure 5 Association of changes in food subgroups with changes in plasma phospholipid fatty acid groups from 1993-1997 to 1998-2000. Mixed-effects linear regression models were used in the analyses. Values indicate an average relative difference in changes/year in mol% of each plasma fatty acid group between 1993-1997 to 1998-2000 per standard serving/day/year increase in each dairy or vegetable subgroup between the same time period (* p<0.05 and ** p<0.01). Serving sizes were defined as 100g/d for low-fat dairy, high-fat dairy, milk, yogurt, cheese, starchy and non-starchy vegetable; as 10g/d for butter, other dairy and avocado. Values in blue font represent negative associations; values in red font represent positive associations. Other dairy included single/double cream, dairy desserts (ex. dairy in other recipes. SFA, saturated fatty acids; PUFA, polyunsaturated fatty acids; MFUA, monounsaturated fatty acids; TFA, trans fatty acids.

Supplemental Figure 6 Change of BMI and physical activity and with change of fatty acid groups. Mixed-effects linear regression models were used in the analyses. **(A)** represents an average relative difference (%) in changes/year in mol% of each fatty acid group, per 1 unit (kg/m^2) change in BMI between 1993-1997 to 1998-2000, adjusting for potential confounders; **(B)** represents an average relative difference (%) in changes/year in mol% of each fatty acid group, per 1-category change of physical activity status between 1993-1997 to 2004-2011, adjusting for potential confounders. SFA, saturated fatty acids; PUFA, polyunsaturated fatty acids; MFUA, monounsaturated fatty acids; TFA, trans fatty acids. *p=0.047 for the association between change in BMI and change in even-chain SFA.

Supplemental Table 1 Food item components of food groups

**Food groups**	Disaggregation from composite dishes	Food compositions
Total Fruit	Yes	Apples, pears, oranges/satsumas/mandarins, grapefruit, bananas, grapes, melon, peaches/plums/apricots, strawberries/raspberries/kiwi fruit, tinned fruit, dried fruit, and fruit used in dishes such as stewed fruit, fruit crumble and fruit fool, for which a percentage of fruit content is assigned (fruit juice, fruit pure´e in yogurt and jams are set to 0% fruit)
Total vegetable	Yes	Parsnips/turnips/swede, sweetcorn, peas, carrots, spinach, broccoli/spring greens/kale, Brussels sprouts, cabbage, green/broad/runner/French beans, courgette/marrow, cauliflower, leeks, onions, garlic, mushrooms, sweet peppers, beansprouts, green salad, watercress, tomatoes, beetroot, coleslaw, avocado, and vegetables used in dishes, for which a percentage of vegetable content is assigned (potatoes, lentils/tofu/baked beans are not considered vegetables)
Legume	No	Tofu, soya meat, TVP, vegeburger; dried lentils/beans/peas; baked beans
Total dairy	Yes	Milk, cheese (hard, soft, cottage cheese), yogurt (low-fat, full-fat, fromage frais), butter, other dairy (single/double cream, dairy desserts), and dairy used in dishes, for which a percentage of dairy content is assigned
Total eggs	Yes	Whole egg, egg used in dishes, for which a percentage of egg content is assigned (fruit cake, frozen trifle, cheese quiche/flan, quiche Lorraine, coleslaw, potato salad, mayonnaise, salad cream, moussaka, croissants, sponge cake and pudding)
White fish	Yes	Fish in batter (as in fish and chips), fish cake and fish finger, other white fish, taramasalata
Fatty fish	Yes	Fresh or canned oily fish (eg, mackerel, kippers, tuna, salmon, sardines, herring)
Red meat	Yes	Beef, beefburgers, pork, lamb, and red meat used in dishes, for which a percentage of red meat content is assigned (Lasagne, moussaka, savoury pies), offal is excluded
White meat	Yes	Chicken, turkey and white meat used in dishes, for which a percentage of white meat content is assigned (meat soup)
Processed meat	Yes	Bacon, ham, corned beef, luncheon meat, sausages, and processed meat used in dishes, for which a percentage of processed meat content is assigned (Quiche, liver sausage)
Liver	No	Liver, liver pate, liver sausage
Potatoes	No	Potatoes, roast, boiled, chips (includes crisps & snacks)
Cereal	No	Rice, pasta, pizza, pies/quiche/sponge puddings/dairy-based puddings/biscuits & crackers, breakfast cereals & porridge
Bread	No	Bread & rolls (white, brown & wholemeal)
Sweets	No	Sweet biscuits (chocolate or plain), cakes (homemade or commercial), buns, pastries (homemade or commercial), fruit pies, tarts, crumbles (homemade or commercial), sponge pudding (homemade or commercial), milk puddings; ice cream, choc ices; chocolates (singles or squares), chocolate snack bars, sweets, toffees, mints
Nuts and seeds	No	Nuts and peanut butter
Tea	No	Tea
Coffee	No	Coffee, instant or ground
Fruit juice	No	Pure fruit juice (100%), eg orange, apple
Sugar-sweetened beverage	No	Fizzy soft drinks, low calorie or diet; Fizzy soft drinks, e.g. Coca Cola, lemonade; Fruit squash or cordial
Alcohol	No	Alcohol consumption (current)

Margarine	No	Polyunsaturated & soft margarines & spreads including low & very-low fat
Vegetable oil	No	Vegetable oil (based on fat specified for baking/cooking & recipes consumed)
**Dairy and vegetable subgroups**	No	
Low-fat dairy	No	Yogurt (low fat, full-fat, fromage frais), semi-skimmed milk, skimmed milk, calcium-fortified milk (0.5%fat), non-specific milk (similar to semi-skimmed)
High-fat dairy	No	Cheese (hard, soft, cottage cheese), butter, cream (single or sour), cream (double or clotted), full-fat milk, Channel Islands/gold milk (5.1%fat), sheep's milk (assumed to be full fat), goat's milk (assumed to be full fat), evaporated/unsweetened condensed milk (9.4%fat), evaporated milk whole diluted
Milk	No	Milk (of dairy origin; ex. milk in puddings/custard/other recipes)
Yogurt	No	Yogurt (low-fat, full-fat, fromage frais)
Cheese	No	Cheese (hard, soft, cottage cheese; ex. cheese in other dishes/recipes)
Other dairy	No	Other dairy (single/double cream, dairy desserts (ex. dairy in other recipes)
Butter	No	Butter
Starchy vegetable	No	Parsnips/turnips/swede, sweetcorn, potatoes, peas
Non-starchy vegetable	No	Carrots, spinach, broccoli/spring greens/kale, Brussels sprouts, cabbage, green/broad/runner/French beans, courgette/marrow, cauliflower, leeks, onions, garlic, mushrooms, sweet peppers, beansprouts, green salad, watercress, tomatoes, beetroot, coleslaw
Avocado	No	Avocado

Supplemental Table 2 Distribution and change of food group consumption from 1993-1997 to 1998-2000 in the EPIC-Norfolk study

Food groups	n	Health Check 1[1] (1993-1997)		Health Check 2[1] (1998-2000)		Change between health check 1 and 2	
		Mean (SD), g/d[2]	Mean (SD), serving/d	Mean (SD), g/d	Mean (SD), serving/d	Mean (SD), g/d	Mean (SD), serving/d
Total Fruit	596	264.4 (160.3)	2.64 (1.6)	287.2 (170.2)	2.87 (1.7)	22.8 (137.2)	0.23 (1.37)
Total vegetable	596	268.1 (111.7)	2.68 (1.12)	258.8 (115.1)	2.59 (1.15)	-9.3 (96.1)	-0.09 (0.96)
Legume	607	22.7 (22.1)	0.23 (0.22)	20.6 (20.2)	0.21 (0.2)	-2 (20.5)	-0.02 (0.21)
Total dairy	596	443.4 (173.3)	4.43 (1.73)	428.2 (171)	4.28 (1.71)	-15.2 (147.6)	-0.15 (1.48)
Total eggs	596	18.2 (12.4)	0.18 (0.12)	17.3 (12.2)	0.17 (0.12)	-0.9 (11.8)	-0.01 (0.12)
White fish	598	17.7 (12)	0.18 (0.12)	17.3 (12.5)	0.17 (0.13)	-0.3 (12.6)	0 (0.13)
Fatty fish	598	13.3 (14.3)	0.13 (0.14)	13.2 (14.4)	0.13 (0.14)	-0.1 (15.9)	0 (0.16)
Red meat	598	37 (24.8)	0.37 (0.25)	35.3 (25.5)	0.35 (0.25)	-1.6 (24.4)	-0.02 (0.24)
White meat	598	26.5 (18.9)	0.27 (0.19)	28.4 (20.3)	0.28 (0.2)	1.9 (20.1)	0.02 (0.2)
Processed meat	598	17.8 (13.6)	0.18 (0.14)	18.1 (14.4)	0.18 (0.14)	0.3 (13.5)	0 (0.13)
Liver	601	1.8 (2.8)	0.18 (0.28)	1.5 (2.6)	0.15 (0.26)	-0.4 (3)	-0.04 (0.3)
Potatoes	598	115.4 (51.3)	1.15 (0.51)	110.1 (49.5)	1.1 (0.49)	-5.3 (52.1)	-0.05 (0.52)
Cereal	598	135 (71.9)	1.35 (0.72)	131 (68.7)	1.31 (0.69)	-4 (67)	-0.04 (0.67)
Bread	598	76.1 (50.1)	0.76 (0.5)	64.9 (45.9)	0.65 (0.46)	-11.2 (49.3)	-0.11 (0.49)
Sweets	610	121.1 (89.2)	1.21 (0.89)	112.1 (85.1)	1.12 (0.85)	-9 (66.5)	-0.09 (0.66)
Nuts and seeds	598	3.3 (6.1)	0.33 (0.61)	3 (6.1)	0.3 (0.61)	-0.3 (6.2)	-0.03 (0.62)
Tea	609	610.1 (369.4)	6.1 (3.69)	611.1 (367.6)	6.11 (3.68)	1 (243.1)	0.01 (2.43)
Coffee	604	327.9 (318.2)	3.28 (3.18)	313.3 (297.7)	3.13 (2.98)	-14.6 (254.3)	-0.15 (2.54)
Fruit juice	608	54.2 (57.9)	0.54 (0.58)	56 (61)	0.56 (0.61)	1.8 (55.5)	0.02 (0.56)
Sugar-sweetened beverage	609	69.6 (99.3)	0.7 (0.99)	70.6 (103.4)	0.71 (1.03)	1 (91.9)	0.01 (0.92)
Alcohol	711	6.7 (7.1)	0.67 (0.71)	6.7 (7.1)	0.67 (0.71)	0 (3.8)	0 (0.38)
Margarine	596	15.2 (14.7)	1.52 (1.47)	13.6 (13.8)	1.36 (1.38)	-1.6 (15.2)	-0.16 (1.52)
Vegetable oil	596	0.3 (0.9)	0.29 (0.91)	0.4 (1)	0.37 (1)	0.1 (1.3)	0.08 (1.31)
**Dairy and vegetable subgroups**							
Low-fat dairy	611	335.5 (203.9)	3.36 (2.04)	336 (196.3)	3.36 (1.96)	0.4 (170.3)	0 (1.7)
High-fat dairy	611	77.8 (134.2)	0.78 (1.34)	64.3 (120.9)	0.64 (1.21)	-13.5 (93.6)	-0.14 (0.94)
Milk	611	347.6 (159.8)	3.48 (1.6)	337 (162.4)	3.37 (1.62)	-10.6 (137)	-0.11 (1.37)
Yogurt	599	44.5 (48)	0.44 (0.48)	44 (46.2)	0.44 (0.46)	-0.5 (42.4)	-0.01 (0.42)
Cheese	602	19.2 (14.9)	0.19 (0.15)	17.2 (14.3)	0.17 (0.14)	-2 (14.6)	-0.02 (0.15)
Butter	601	3.9 (8.2)	0.39 (0.82)	3.8 (7.7)	0.38 (0.77)	-0.1 (6.5)	-0.01 (0.65)
Other dairy	597	4.5 (7.8)	0.45 (0.78)	4.8 (8.8)	0.48 (0.88)	0.3 (8.9)	0.03 (0.89)
Starchy vegetable	722	144.9 (64.9)	1.45 (0.65)	117.9 (73.8)	1.18 (0.74)	-27 (80.4)	-0.27 (0.8)
Non-starchy vegetable	722	210.7 (101.8)	2.11 (1.02)	177.1 (119.2)	1.77 (1.19)	-33.6 (115.6)	-0.34 (1.16)
Avocado	596	1.4 (3.2)	0.14 (0.32)	1.5 (3.3)	0.15 (0.33)	0.1 (2.6)	0.01 (0.26)

[1] Health check 1 represents time period of the baseline recruitment (1993-1997); these participants were followed up 3 to 4 years after recruitment (health check 2, 1998-2000), and on average 13 years after recruitment (health check 3, 2004-2011)

[2] The unit was g/d for all the dietary factors, except for alcohol (unit/wk was used).

Supplemental Table 3 Intraclass correlation coefficient for the repeated measure of plasma phospholipid fatty acids across the three time points from 1993-1997 to 2004-2011 [1]

	Intraclass correlation coefficient (95%CI)
Odd-chain SFA	0.48 (0.43, 0.52)
C15:0	0.48 (0.43, 0.52)
C17:0	0.40 (0.36, 0.45)
Even-chain SFA	0.24 (0.19, 0.29)
C14:0	0.30 (0.25, 0.35)
C16:0	0.41 (0.36, 0.45)
C18:0	0.41 (0.36, 0.45)
Very-long-chain SFA	0.21 (0.16, 0.26)
C20:0	0.15 (0.11, 0.20)
C22:0	0.20 (0.15, 0.25)
C23:0	0.18 (0.13, 0.23)
C24:0	0.28 (0.24, 0.33)
Plant n-3 PUFA (C18:3n3)	0.14 (0.09, 0.19)
Marine n-3 PUFA	0.45 (0.40, 0.49)
C20:5n3	0.38 (0.33, 0.42)
C22:5n3	0.41 (0.36, 0.46)
C22:6n3	0.49 (0.45, 0.53)
n-6 PUFA	0.41 (0.36, 0.45)
C18:2n6c	0.49 (0.45, 0.53)
C18:3n6	0.34 (0.30, 0.39)
C20:2n6	0.45 (0.40, 0.49)
C20:3n6	0.54 (0.50, 0.58)
C20:4n6 + C20:3n3	0.60 (0.56, 0.63)
C22:4n6	0.51 (0.47, 0.56)
C22:5n6	0.47 (0.43, 0.52)
MUFA	0.38 (0.33, 0.42)
C16:1	0.54 (0.50, 0.58)
C17:1	0.09 (0.05, 0.15)
C18:1n9c	0.36 (0.31, 0.41)
C20:1n9	0.20 (0.15, 0.25)
C24:1	0.26 (0.21, 0.31)
TFA	0.26 (0.22, 0.32)
C18:1n9t	0.27 (0.22, 0.32)
C18:2n6t	0.17 (0.12, 0.22)

[1]Intraclass correlation coefficient (ICC) values represent proportion of total variance that is due to variation between individuals, from a 2-level model: level 1 (measurement occasion), level 2 (individual), adjusting for time since baseline for each of health-check 2 and health-check 3. SFA, saturated fatty acids; PUFA, polyunsaturated fatty acids; MFUA, monounsaturated fatty acids; TFA, trans fatty acids.

Supplemental Table 4 Association of changes in food groups with changes in plasma phospholipid fatty acid groups from 1993-1997 to 1998-2000

Food groups	Annual percentage change (95% confidence intervals) in fatty acid group[1]							
	Odd-chain SFA	Even-chain SFA	Very-long-chain SFA	Plant n-3 PUFA	Marine n-3 PUFA	n-6 PUFA	MUFA	TFA
Annual % change (95%CI)	-0.63 (-0.73, -0.52)	-0.05 (-0.06, -0.03)	0.03 (-0.11, 0.16)	-0.23 (-0.51, 0.05)	1.28 (1.11, 1.44)	-0.25 (-0.29, -0.2)	0.45 (0.37, 0.54)	-7.84 (-8.07, -7.61)
Total fruit	-0.38 (-1.53, 0.77)	0 (-0.14, 0.14)	-0.64 (-1.60, 0.34)	0.56 (-2.06, 3.25)	-0.97 (-2.47, 0.55)	0.13 (-0.29, 0.56)	0.02 (-0.75, 0.80)	-0.21 (-2.60, 2.24)
Total vegetable	0.23 (-1.47, 1.97)	-0.02 (-0.23, 0.19)	-0.96 (-2.39, 0.48)	-0.42 (-4.26, 3.58)	2.71 (0.41, 5.06)	-0.33 (-0.96, 0.30)	-0.9 (-2.04, 0.25)	0.97 (-2.62, 4.69)
Legume	6.18 (-1.92, 15.0)	0.73 (-0.23, 1.71)	-7.64 (-13.6, -1.23)	18.9 (-0.93, 42.6)	-4.24 (-13.8, 6.36)	-1.15 (-4.00, 1.78)	-0.89 (-6.05, 4.56)	-8.36 (-22.49, 8.36)
Total dairy	-0.64 (-1.70, 0.44)	0 (-0.13, 0.13)	0.05 (-0.86, 0.97)	0.26 (-2.20, 2.79)	-0.25 (-1.66, 1.18)	0.03 (-0.37, 0.43)	0.50 (-0.23, 1.24)	-0.01 (-2.26, 2.30)
Eggs	-6.93 (-20.1, 8.40)	-2.71 (-4.5, -0.90)	6.11 (-6.75, 20.8)	-24.2 (-46.7, 7.67)	-4.15 (-21.7, 17.3)	5.11 (-0.65, 11.2)	3.26 (-6.85, 14.5)	27.5 (-7.69, 76.0)
White fish	-9.22 (-20.0, 2.97)	0.36 (-1.16, 1.90)	6.07 (-4.63, 18.0)	-13.1 (-34.9, 16.1)	-6.44 (-20.8, 10.5)	4.89 (0.13, 9.86)	-11.2 (-18.4, -3.29)	7.29 (-17.8, 40.0)
Fatty fish	-7.48 (-16.4, 2.39)	-0.54 (-1.75, 0.69)	0.62 (-7.63, 9.61)	10.62 (-12.3, 39.6)	19.3 (4.43, 36.3)	-3.42 (-6.95, 0.24)	-2.35 (-8.79, 4.53)	22.1 (-1.34, 51.1)
Red meat	-2.61 (-8.98, 4.21)	-0.11 (-0.93, 0.71)	-0.76 (-6.27, 5.08)	-10.8 (-23.6, 4.2)	3.62 (-5.25, 13.3)	0.40 (-2.07, 2.93)	-2.77 (-7.1, 1.76)	9.48 (-5.08, 26.3)
White meat	-9.34 (-16.3, -1.81)	-0.23 (-1.19, 0.73)	-8.25 (-14.2, -1.85)	-2.26 (-18.6, 17.4)	7.92 (-2.88, 19.9)	0.18 (-2.72, 3.16)	-2.39 (-7.50, 3.00)	6.34 (-10.1, 25.8)
Processed meat	13.0 (-0.19, 27.9)	0.09 (-1.40, 1.60)	5.12 (-5.32, 16.7)	-0.27 (-25.0, 32.6)	-0.03 (-15.1, 17.8)	-0.16 (-4.61, 4.50)	0.81 (-7.27, 9.59)	-23.6 (-41.2, -0.77)
Liver	2.80 (-2.54, 8.44)	-0.05 (-0.69, 0.60)	-1.68 (-6.01, 2.86)	-10.6 (-20.87, 1.11)	-2.09 (-8.76, 5.06)	-0.04 (-1.99, 1.94)	1.97 (-1.63, 5.70)	4.50 (-6.63, 16.96)
Potatoes	-2.60 (-5.52, 0.41)	0 (-0.37, 0.37)	2.68 (0.08, 5.36)	1.47 (-5.38, 8.81)	-2.42 (-6.27, 1.58)	0.19 (-0.93, 1.31)	1.10 (-0.95, 3.19)	-2.33 (-8.40, 4.15)
Cereal	1.10 (-1.42, 3.69)	0.40 (0.09, 0.71)	-0.14 (-2.25, 2.01)	0.22 (-5.42, 6.20)	-4.16 (-7.31, -0.91)	0.53 (-0.40, 1.47)	0.27 (-1.42, 1.99)	-5.4 (-10.3, -0.22)
Bread	1.07 (-2.32, 4.58)	0.08 (-0.33, 0.50)	-3.30 (-6.05, -0.47)	1.78 (-5.89, 10.08)	-1.75 (-6.08, 2.79)	-0.02 (-1.27, 1.24)	1.59 (-0.72, 3.95)	0.69 (-6.31, 8.21)
Sweets	0.34 (-2.46, 3.21)	-0.18 (-0.52, 0.16)	-1.70 (-4.02, 0.66)	2.83 (-3.63, 9.71)	1.39 (-2.32, 5.24)	-0.38 (-1.41, 0.66)	0.16 (-1.72, 2.09)	1.34 (-4.50, 7.54)
Nuts and seeds	1.37 (-1.19, 4.00)	-0.08 (-0.39, 0.23)	2.33 (0.15, 4.55)	1.11 (-4.65, 7.21)	-0.16 (-3.46, 3.26)	0.23 (-0.70, 1.18)	-1.06 (-2.75, 0.66)	-4.22 (-9.25, 1.08)
Tea	-0.06 (-0.72, 0.61)	0.06 (-0.02, 0.14)	-0.11 (-0.67, 0.45)	-0.08 (-1.59, 1.45)	-0.50 (-1.37, 0.37)	-0.01 (-0.25, 0.24)	0.04 (-0.40, 0.49)	0.59 (-0.81, 2.00)
Coffee	-0.16 (-0.77, 0.45)	-0.05 (-0.13, 0.02)	0.14 (-0.38, 0.66)	0.22 (-1.19, 1.64)	0.28 (-0.53, 1.10)	-0.09 (-0.32, 0.14)	0.37 (-0.04, 0.79)	-0.15 (-1.44, 1.15)
Fruit juice	-0.85 (-3.74, 2.12)	0.14 (-0.22, 0.50)	-1.01 (-3.44, 1.49)	-2.80 (-9.16, 4.02)	3.12 (-0.82, 7.21)	-0.21 (-1.28, 0.88)	-1.36 (-3.30, 0.62)	-6.46 (-12.1, -0.45)
Sugar-sweetened beverages	1.60 (-0.15, 3.37)	0.04 (-0.17, 0.25)	0.96 (-0.50, 2.45)	0.08 (-3.83, 4.14)	-1.32 (-3.56, 0.97)	0.12 (-0.52, 0.76)	0.21 (-0.95, 1.39)	-4.37 (-7.80, -0.82)
Alcohol	-3.92 (-7.75, 0.07)	0.39 (-0.10, 0.88)	0.68 (-2.72, 4.20)	0.04 (-8.88, 9.83)	4.24 (-1.22, 10.0)	-0.98 (-2.45, 0.52)	1.05 (-1.68, 3.85)	-2.12 (-10.2, 6.65)
Margarine	-1.38 (-2.47, -0.29)	-0.04 (-0.17, 0.10)	-0.43 (-1.36, 0.51)	-1.40 (-3.88, 1.15)	-0.86 (-2.30, 0.61)	0.52 (0.12, 0.93)	-0.91 (-1.65, -0.17)	0.93 (-1.41, 3.32)
Vegetable oil	0.80 (-0.40, 2.02)	-0.03 (-0.17, 0.12)	-1.35 (-2.35, -0.35)	0.37 (-2.35, 3.16)	-0.78 (-2.34, 0.80)	0.43 (-0.01, 0.88)	-0.77 (-1.56, 0.04)	-0.20 (-2.69, 2.35)

[1] Mixed-effects linear regression models were used in the analyses. Values (95%CI) represent an annual relative difference (%) in changes in mol% of these fatty acid groups per 1-standard serving /day/year increase in food groups between the first two time points (from 1993-1997 to 1998-2000), adjusting for participant recruitment year, sex, follow-up duration, BMI change between the two time points, and baseline variables of age, BMI, physical activity, smoking status, educational level, alcohol drinking, social class, total energy intake, fish oil supplements, corresponding fatty acid group and food group, and mutual adjustment for the change in all the other food groups and their interactions with follow-up duration.

Serving sizes were defined as 100g/d for fruit, vegetable, legume, dairy, eggs, white fish, fatty fish, red meat, white meat, processed meat, potatoes, cereal, bread, tea, coffee, fruit juice, sugar-sweetened beverages, sweets; as 10g/d for margarine, liver, nuts and seeds; as 1g/d for vegetable oil and 10 units/wk for alcohol. SFA, saturated fatty acids; PUFA, polyunsaturated fatty acids; MFUA, monounsaturated fatty acids; TFA, trans fatty acids.

Supplemental Table 5 Association of changes in food groups with changes in plasma phospholipid fatty acid groups in the prevalent change and lagged-change models

Food groups	Model	Annual percentage changes (95% confidence intervals) in fatty acid group							
		Odd-chain SFA	Even-chain SFA	Very-long-chain SFA	Plant n-3 PUFA	Marine n-3 PUFA	n-6 PUFA	MUFA	TFA
	Annual % change (95%CI)	-0.63 (-0.73, -0.52)	-0.05 (-0.06, -0.03)	0.03 (-0.11, 0.16)	-0.23 (-0.51, 0.05)	1.28 (1.11, 1.44)	-0.25 (-0.29, -0.2)	0.45 (0.37, 0.54)	-7.84 (-8.07, -7.61)
Total fruit	Prevalent change[1]	-0.03 (-0.10, 0.04)	0 (-0.01, 0.01)	-0.04 (-0.13, 0.05)	0.16 (-0.03, 0.35)	-0.01 (-0.12, 0.1)	-0.02 (-0.05, 0.02)	0.03 (-0.03, 0.09)	0.01 (-0.16, 0.17)
	Lagged-change[2]	0.09 (-0.34, 0.51)	-0.03 (-0.08, 0.03)	0.55 (0.11, 0.98)	-0.68 (-1.67, 0.32)	0.40 (-0.2, 0.99)	-0.03 (-0.20, 0.15)	0.02 (-0.29, 0.33)	0.35 (-0.58, 1.29)
Total vegetable	Prevalent change	0.07 (-0.03, 0.18)	-0.01 (-0.03, 0)	0.20 (0.07, 0.34)	-0.10 (-0.38, 0.19)	-0.02 (-0.19, 0.15)	0.02 (-0.03, 0.07)	-0.02 (-0.11, 0.07)	0.03 (-0.22, 0.28)
	Lagged-change	-0.14 (-0.77, 0.49)	0.01 (-0.06, 0.09)	-0.92 (-1.56, -0.28)	-0.16 (-1.64, 1.35)	-0.16 (-1.04, 0.73)	0.06 (-0.20, 0.32)	0.26 (-0.20, 0.72)	-0.53 (-1.91, 0.86)
Legume	Prevalent change	0.23 (-0.26, 0.72)	0.01 (-0.07, 0.08)	0.21 (-0.41, 0.84)	-0.70 (-20, 0.62)	1.22 (0.44, 2.01)	-0.25 (-0.47, -0.03)	0.32 (-0.09, 0.73)	-1.31 (-2.43, -0.18)
	Lagged-change	-0.74 (-3.63, 2.23)	0.14 (-0.22, 0.50)	2.25 (-0.80, 5.40)	0.22 (-6.56, 7.49)	-1.90 (-5.87, 2.24)	-0.06 (-1.27, 1.16)	0.01 (-2.13, 2.19)	4.45 (-2.13, 11.46)
Total dairy	Prevalent change	-0.05 (-0.11, 0.02)	0.01 (0, 0.02)	-0.02 (-0.10, 0.06)	-0.07 (-0.24, 0.10)	0.04 (-0.06, 0.14)	-0.01 (-0.04, 0.02)	-0.04 (-0.09, 0.01)	0.07 (-0.08, 0.22)
	Lagged-change	0.09 (-0.31, 0.50)	0 (-0.05, 0.05)	0.30 (-0.11, 0.72)	-0.35 (-1.31, 0.62)	-0.23 (-0.79, 0.34)	0.05 (-0.12, 0.22)	-0.05 (-0.35, 0.24)	0.08 (-0.82, 0.98)
Eggs	Prevalent change	-0.84 (-1.83, 0.16)	0.16 (0, 0.32)	0.10 (-1.17, 1.39)	1.50 (-1.21, 4.28)	-2.10 (-3.65, -0.53)	0.39 (-0.07, 0.86)	-0.77 (-1.59, 0.06)	1.73 (-0.62, 4.14)
	Lagged-change	3.14 (-2.65, 9.27)	-0.03 (-0.72, 0.68)	-0.54 (-6.26, 5.53)	-5.21 (-17.3, 8.67)	0.58 (-7.23, 9.06)	-0.20 (-2.55, 2.21)	0.29 (-3.86, 4.61)	1.28 (-10.76, 14.95)
White fish	Prevalent change	0.16 (-0.79, 1.11)	-0.04 (-0.18, 0.11)	-0.34 (-1.52, 0.85)	0.86 (-1.66, 3.44)	0.01 (-1.47, 1.51)	-0.23 (-0.66, 0.20)	0.16 (-0.62, 0.95)	1.91 (-0.30, 4.16)
	Lagged-change	0.20 (-4.51, 5.15)	0.13 (-0.46, 0.71)	0.20 (-4.64, 5.27)	-3.99 (-14.4, 7.61)	-1.57 (-7.99, 5.3)	0.88 (-1.11, 2.90)	0.63 (-2.86, 4.23)	0.99 (-9.15, 12.3)
Fatty fish	Prevalent change	-0.37 (-1.16, 0.42)	-0.07 (-0.19, 0.05)	0.08 (-0.92, 1.09)	1.40 (-0.74, 3.58)	-1.70 (-2.93, -0.46)	0.25 (-0.11, 0.61)	0.39 (-0.26, 1.05)	0.98 (-0.87, 2.86)
	Lagged-change	0.24 (-3.46, 4.08)	0.21 (-0.24, 0.67)	-1.25 (-4.99, 2.63)	-6.16 (-14.17, 2.59)	-1.61 (-6.64, 3.70)	0.60 (-0.95, 2.17)	-0.78 (-3.46, 1.99)	-0.22 (-8.13, 8.38)
Red meat	Prevalent change	0.05 (-0.42, 0.53)	0.04 (-0.04, 0.11)	-0.18 (-0.78, 0.43)	0.48 (-0.80, 1.77)	-0.17 (-0.92, 0.59)	0.04 (-0.18, 0.26)	-0.12 (-0.51, 0.28)	0.19 (-0.92, 1.31)
	Lagged-change	0.40 (-2.07, 2.93)	-0.04 (-0.34, 0.26)	0.91 (-1.64, 3.52)	-0.55 (-6.26, 5.51)	2.06 (-1.43, 5.69)	-0.31 (-1.33, 0.72)	0.24 (-1.56, 2.09)	0.87 (-4.52, 6.56)
White meat	Prevalent change	0.31 (-0.24, 0.87)	0.06 (-0.02, 0.15)	0.17 (-0.53, 0.87)	-0.55 (-2.02, 0.94)	0.14 (-0.73, 1.02)	-0.28 (-0.53, -0.03)	0.48 (0.03, 0.95)	-0.12 (-1.39, 1.18)
	Lagged-change	2.78 (-0.18, 5.83)	-0.15 (-0.51, 0.2)	0.65 (-2.33, 3.72)	-2.54 (-9.08, 4.48)	0.51 (-3.52, 4.71)	0.32 (-0.89, 1.54)	-0.73 (-2.83, 1.42)	-4.35 (-10.3, 2.03)
Processed meat	Prevalent change	-0.82 (-1.68, 0.06)	0.03 (-0.11, 0.17)	-1.14 (-2.23, -0.04)	1.50 (-0.86, 3.92)	0.02 (-1.36, 1.42)	-0.02 (-0.42, 0.38)	0.26 (-0.47, 0.99)	-0.1 (-2.11, 1.96)
	Lagged-change	-3.19 (-7.55, 1.37)	0.20 (-0.36, 0.76)	-1.74 (-6.26, 3.01)	-2.36 (-12.5, 8.91)	-1.68 (-7.82, 4.87)	0.28 (-1.60, 2.21)	-0.84 (-4.12, 2.56)	-0.46 (-10.0, 10.2)
Liver	Prevalent change	0.08 (-0.31, 0.47)	0.01 (-0.05, 0.08)	-0.06 (-0.55, 0.44)	-0.07 (-1.11, 0.98)	0.07 (-0.55, 0.69)	-0.10 (-0.28, 0.08)	0.22 (-0.11, 0.54)	0.36 (-0.55, 1.27)
	Lagged-change	-1.90 (-3.87, 0.11)	-0.20 (-0.45, 0.04)	0.58 (-1.50, 2.70)	1.33 (-3.45, 6.35)	-0.89 (-3.66, 1.97)	0.66 (-0.18, 1.51)	-0.78 (-2.24, 0.70)	1.53 (-2.93, 6.19)
Potatoes	Prevalent change	0.07 (-0.16, 0.30)	-0.02 (-0.05, 0.02)	-0.26 (-0.55, 0.03)	-0.14 (-0.75, 0.48)	-0.32 (-0.68, 0.05)	0.09 (-0.02, 0.19)	-0.03 (-0.22, 0.16)	-0.01 (-0.54, 0.53)
	Lagged-change	-0.29 (-1.45, 0.89)	-0.01 (-0.15, 0.13)	0.78 (-0.42, 2.00)	-0.25 (-2.99, 2.56)	0.19 (-1.44, 1.85)	0.14 (-0.34, 0.63)	-0.64 (-1.49, 0.22)	0.8 (-1.77, 3.44)

Cereal	Prevalent change	0.03 (-0.13, 0.19)	-0.03 (-0.05, -0.01)	0.13 (-0.07, 0.33)	-0.03 (-0.45, 0.40)	0.09 (-0.16, 0.34)	0.01 (-0.06, 0.09)	-0.01 (-0.14, 0.12)	0.31 (-0.06, 0.68)
	Lagged-change	-0.03 (-0.93, 0.87)	0.03 (-0.08, 0.14)	-1.05 (-1.96, -0.13)	1.09 (-1.06, 3.28)	0.88 (-0.39, 2.16)	-0.20 (-0.57, 0.17)	0.16 (-0.50, 0.83)	-0.63 (-2.59, 1.37)
Bread	Prevalent change	0.03 (-0.20, 0.26)	-0.02 (-0.05, 0.02)	0.09 (-0.20, 0.39)	0.02 (-0.60, 0.65)	0.07 (-0.30, 0.44)	-0.01 (-0.11, 0.10)	0.09 (-0.10, 0.28)	-0.03 (-0.56, 0.52)
	Lagged-change	-0.67 (-1.95, 0.62)	-0.12 (-0.27, 0.04)	-0.35 (-1.66, 0.98)	0.15 (-2.88, 3.26)	-0.23 (-2.01, 1.59)	0.46 (-0.07, 1.00)	-0.37 (-1.30, 0.58)	1.31 (-1.53, 4.23)
Sweets	Prevalent change	-0.05 (-0.21, 0.11)	0.01 (-0.01, 0.04)	-0.01 (-0.21, 0.19)	-0.02 (-0.44, 0.41)	0.01 (-0.24, 0.26)	0.02 (-0.05, 0.09)	-0.01 (-0.15, 0.12)	-0.96 (-1.32, -0.59)
	Lagged-change	-0.34 (-1.40, 0.74)	0.1 (-0.03, 0.23)	0.69 (-0.41, 1.81)	0.69 (-1.84, 3.30)	-0.23 (-1.72, 1.29)	-0.15 (-0.59, 0.30)	-0.07 (-0.86, 0.72)	0.05 (-2.29, 2.45)
Nuts and seeds	Prevalent change	-0.04 (-0.21, 0.14)	0.04 (0.01, 0.07)	-0.17 (-0.39, 0.05)	0.13 (-0.33, 0.60)	0.19 (-0.09, 0.46)	-0.1 (-0.18, -0.02)	0.05 (-0.10, 0.19)	0.06 (-0.34, 0.46)
	Lagged-change	0.31 (-0.70, 1.34)	-0.05 (-0.17, 0.07)	0.61 (-0.43, 1.67)	-0.9 (-3.26, 1.52)	0.34 (-1.08, 1.78)	-0.03 (-0.45, 0.39)	0.16 (-0.59, 0.90)	0.8 (-1.43, 3.08)
Tea	Prevalent change	0 (-0.04, 0.03)	0 (0, 0.01)	0.03 (-0.01, 0.07)	-0.03 (-0.11, 0.06)	0 (-0.05, 0.05)	-0.01 (-0.02, 0.01)	-0.01 (-0.04, 0.01)	0.03 (-0.04, 0.10)
	Lagged-change	-0.02 (-0.26, 0.23)	-0.02 (-0.05, 0.01)	-0.09 (-0.34, 0.16)	0 (-0.58, 0.57)	0.12 (-0.22, 0.46)	-0.02 (-0.12, 0.08)	0.13 (-0.05, 0.31)	-0.02 (-0.55, 0.52)
Coffee	Prevalent change	0 (-0.04, 0.03)	0 (0, 0.01)	-0.01 (-0.06, 0.04)	0.01 (-0.08, 0.11)	-0.04 (-0.10, 0.01)	0 (-0.02, 0.01)	0.01 (-0.02, 0.04)	0.03 (-0.05, 0.12)
	Lagged-change	0.15 (-0.07, 0.38)	0.01 (-0.02, 0.04)	-0.05 (-0.27, 0.18)	-0.2 (-0.72, 0.33)	0.10 (-0.21, 0.41)	0 (-0.09, 0.09)	-0.13 (-0.29, 0.03)	-0.02 (-0.51, 0.47)
Fruit juice	Prevalent change	0.09 (-0.09, 0.28)	-0.01 (-0.04, 0.01)	0 (-0.23, 0.24)	-0.04 (-0.54, 0.46)	-0.11 (-0.41, 0.18)	0 (-0.08, 0.09)	0.10 (-0.06, 0.25)	0.16 (-0.27, 0.60)
	Lagged-change	0.36 (-0.71, 1.45)	0.03 (-0.10, 0.16)	0.35 (-0.75, 1.46)	0.59 (-1.94, 3.18)	-0.84 (-2.32, 0.67)	-0.11 (-0.55, 0.33)	0.59 (-0.19, 1.39)	2.59 (0.20, 5.04)
Sugar-sweetened beverages	Prevalent change	0.04 (-0.07, 0.14)	0 (-0.02, 0.02)	0.07 (-0.07, 0.20)	-0.26 (-0.55, 0.03)	0.05 (-0.12, 0.22)	0.01 (-0.04, 0.06)	-0.11 (-0.20, -0.02)	-0.09 (-0.34, 0.16)
	Lagged-change	-0.47 (-1.13, 0.19)	0.14 (0.06, 0.22)	-0.53 (-1.20, 0.15)	0.91 (-0.66, 2.51)	0.22 (-0.70, 1.16)	-0.27 (-0.54, 0)	0 (-0.49, 0.48)	0.06 (-1.38, 1.53)
Alcohol	Prevalent change	0.15 (-0.01, 0.30)	-0.04 (-0.06, -0.01)	0 (-0.20, 0.20)	-0.25 (-0.66, 0.17)	0.02 (-0.23, 0.27)	0.05 (-0.02, 0.12)	-0.04 (-0.17, 0.09)	0.54 (0.17, 0.90)
	Lagged-change	-0.07 (-1.59, 1.48)	-0.11 (-0.29, 0.08)	-0.49 (-2.03, 1.09)	2.07 (-1.54, 5.82)	-0.67 (-2.78, 1.49)	0.18 (-0.45, 0.82)	0.33 (-0.78, 1.46)	0.62 (-2.68, 4.03)
Margarine	Prevalent change	0.05 (-0.03, 0.13)	0 (-0.01, 0.01)	-0.05 (-0.15, 0.05)	0.18 (-0.04, 0.39)	0.10 (-0.03, 0.23)	-0.05 (-0.08, -0.01)	0.12 (0.05, 0.19)	-0.26 (-0.45, -0.08)
	Lagged-change	0.44 (0.01, 0.87)	0.04 (-0.01, 0.09)	0.02 (-0.42, 0.46)	-0.09 (-1.10, 0.93)	0.57 (-0.03, 1.17)	-0.11 (-0.28, 0.07)	-0.24 (-0.55, 0.07)	-0.15 (-1.09, 0.79)
Vegetable oil	Prevalent change	0.08 (-0.03, 0.20)	0 (-0.01, 0.02)	0.06 (-0.08, 0.21)	-0.16 (-0.47, 0.14)	0.09 (-0.09, 0.27)	-0.05 (-0.10, 0)	0.07 (-0.02, 0.17)	-0.12 (-0.38, 0.15)
	Lagged-change	-0.16 (-0.59, 0.28)	-0.01 (-0.07, 0.04)	0.09 (-0.35, 0.54)	0.29 (-0.74, 1.33)	-0.17 (-0.78, 0.44)	-0.03 (-0.21, 0.15)	0.28 (-0.04, 0.60)	0.08 (-0.87, 1.05)

[1]**Prevalent change model**: Mixed-effects linear regression models were used in the analyses. Values (95%CI) represent an average relative difference (%) in changes/year in mol% of these fatty acid groups over the 3 time points, per 1-standard serving/day change in baseline food groups, adjusting for age at baseline, sex, BMI at baseline, BMI change between first 2 time points, participant recruitment year, and baseline physical activity, smoking status, educational level, alcohol drinking status (current, former, never), social class, total energy intake, fish oil supplements, follow-up duration and mutual adjustment for the baseline food groups and their interactions with follow-up duration.

[2]**Lagged-change model**: Mixed-effects linear regression models were used in the analyses. Values (95%CI) represent an average relative difference (%) in changes/year in mol% of these fatty acid groups between the second and third time points (from 1998-2000 to 2004-2011) per 1-standard serving/day/year increase in food groups between the first and second time points (from 1993-1997 to 1998-2000), adjusting for age at baseline, sex, BMI at baseline, BMI change between HC1 and HC2, participant recruitment year, and baseline physical activity, smoking status, educational level, alcohol drinking status, social class, total energy intake, fish oil supplements, baseline corresponding fatty acid group and food group, follow-up duration between second and third time points, and mutual adjustment for the change of all the other food groups between the first and second time points and their interactions with follow-up duration between the second and third time points. **Serving sizes** were defined as 100g/d for fruit, vegetable, legume, dairy, eggs, white fish, fatty fish, red meat, white meat, processed meat, potatoes, cereal, bread, tea, coffee, fruit juice, sugar-sweetened beverages, sweets; as 10g/d for margarine, liver, nuts and seeds; as 1g/d for vegetable oil and 10 units/wk for

alcohol. SFA, saturated fatty acids; PUFA, polyunsaturated fatty acids; MFUA, monounsaturated fatty acids; TFA, trans fatty acids.

Supplemental Table 6 Association of baseline age, body mass index, smoking status and physical activity with annual percentage change of plasma phospholipid fatty acid groups[1]

	Odd-chain SFA	Even-chain SFA	Very-long-chain SFA	Plant n-3 PUFA	Marine n-3 PUFA	n-6 PUFA	MUFA	TFA
Age	0.005 (-0.008, 0.018)	-0.003 (-0.005, -0.001)	-0.027 (-0.043, -0.010)	0.020 (-0.014, 0.055)	-0.019 (-0.04, 0.001)	0.008 (0.002, 0.014)	0.003 (-0.008, 0.014)	0.044 (0.013, 0.075)
BMI	0.033 (0.004, 0.063)	-0.001 (-0.005, 0.004)	0.001 (-0.036, 0.039)	-0.012 (-0.09, 0.067)	-0.046 (-0.093, 0.001)	0.002 (-0.012, 0.015)	0.025 (0, 0.050)	-0.020 (-0.091, 0.05)
Current vs never smoker	-0.09 (-0.55, 0.38)	0.01 (-0.06, 0.09)	0.2 (-0.39, 0.79)	-0.41 (-1.63, 0.84)	-0.51 (-1.24, 0.23)	0.18 (-0.03, 0.40)	-0.14 (-0.53, 0.25)	1.09 (-0.02, 2.22)
Former vs never smoker	0.14 (-0.08, 0.36)	-0.03 (-0.06, 0)	-0.02 (-0.30, 0.25)	-0.73 (-1.30, -0.15)	0.14 (-0.21, 0.49)	0.07 (-0.03, 0.17)	-0.08 (-0.27, 0.10)	0.39 (-0.13, 0.91)
Physical activity	0.03 (-0.07, 0.13)	0 (-0.02, 0.01)	0 (-0.12, 0.13)	-0.14 (-0.40, 0.13)	-0.03 (-0.19, 0.12)	0.03 (-0.01, 0.08)	-0.03 (-0.12, 0.05)	-0.21 (-0.45, 0.02)

[1]Mixed-effects linear regression models were used in the analyses. The values are reported as average percentage change (95%CI) of these fatty acids per year over the 3 health checks per 1-y older for age, per 1-unit (kg/m^2) higher for BMI, per 1-category higher (more active) in physical activity, or for current/former smoker vs never smoker, with adjustment for participant recruitment year, sex, baseline variables of educational level, alcohol intake, alcohol drinking, social class, total energy intake and fish oil supplements, follow-up duration, mutual adjustment for the five variables in the table, and baseline intakes of 23 food groups (fruit, vegetable, legume, dairy, eggs, white fish, fatty fish, red meat, white meat, processed meat, potatoes, cereal, bread, tea, coffee, fruit juice, sugar-sweetened beverages, sweets, alcohol, liver, margarine, nuts and seeds, vegetable oil). SFA, saturated fatty acids; PUFA, polyunsaturated fatty acids; MFUA, monounsaturated fatty acids; TFA, trans fatty acids.

Supplemental Figure 1 Flow-chart of the present study.

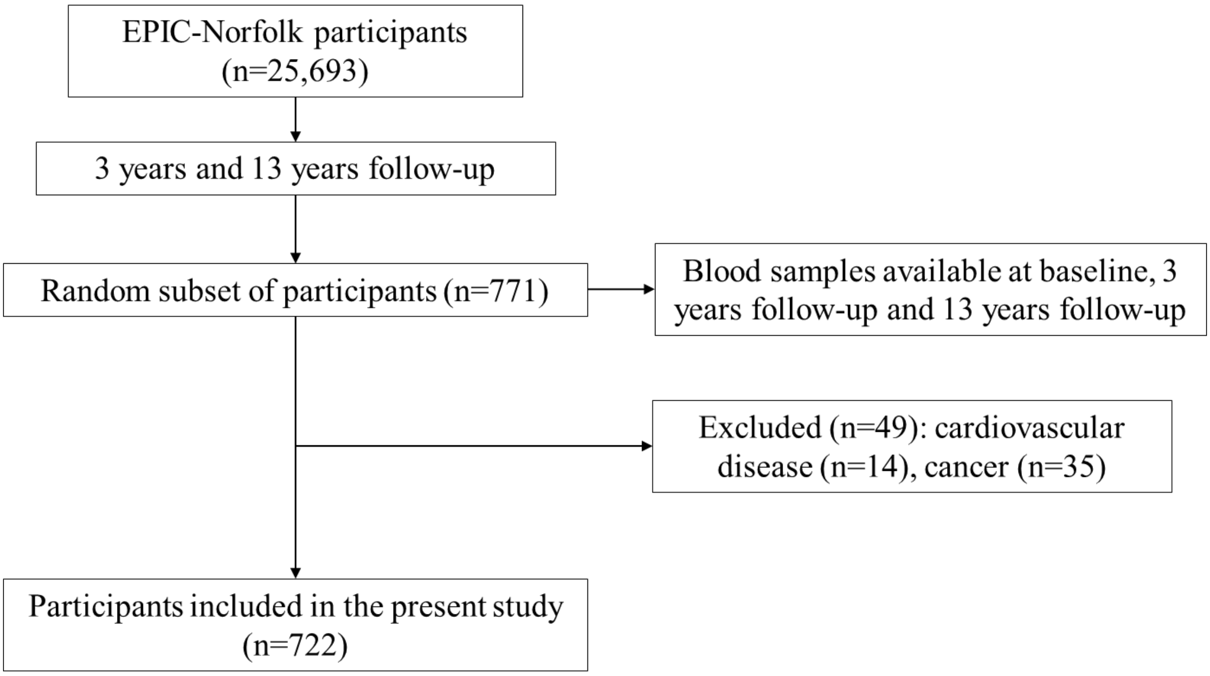

Supplemental Figure 2 Associations between changes in food groups and changes in individual plasma fatty acids from 1993-1997 to 1998-2000. Mixed-effects linear regression models were used in the analyses. Values represent an average relative difference (%) in changes/year in mol% of each individual saturated fatty acids (A), polyunsaturated fatty acids (B), monounsaturated fatty acids and trans fatty acids (C) per one serving/day/year increase in food groups (* p<0.05 and ** p<0.01). Serving sizes were defined as 10 g/d for margarine, liver, nuts and seeds; as 1 g/d for vegetable oil and 10 units/wk for alcohol; and 100 g/day for the other food groups. Red and blue boxes indicate positive and negative associations, respectively, of changes/year in food consumption with changes/year in mol% of fatty acids. Average annual changes in mol% of each fatty acid group are presented on the top. All the estimates were mutually adjusted for changes in food groups and baseline consumption levels of those and adjusted for baseline levels of a fatty acid and other potential confounders (see text in details).

(A) Individual saturated fatty acids

	C15:0	C17:0	C14:0	C16:0	C18:0	C20:0	C22:0	C23:0	C24:0
Annual % changes (95%CI)	-0.74 (-0.87, -0.61)	-0.54 (-0.65, -0.42)	-0.07 (-0.23, 0.08)	-0.02 (-0.05, 0)	-0.08 (-0.13, -0.02)	-0.65 (-0.79, -0.5)	-0.54 (-0.69, -0.38)	0.74 (0.56, 0.92)	0.62 (0.49, 0.76)
Total Fruit	-0.57	-0.19	-0.27	-0.09	0.16	-0.71	-0.81	-0.90	-0.43
Total vegetable	-0.66	0.73	-0.28	0.17	-0.37	-0.89	-0.54	0.71	-1.16
Legume	7.55	5.78	8.16	0.14	3.02	0.10	**-11.8** **	-4.70	**-9.03** *
Total dairy	0.15	-0.96	-0.03	-0.03	-0.05	0.91	-0.55	-0.25	-0.01
Total eggs	-5.10	-9.26	**-23.1** **	-0.90	-5.68	-8.32	13.7	2.07	4.07
White fish	-11.8	-7.17	-13.6	-1.67	4.99	14.2	6.81	**-15.2** *	**13.2** *
Fatty fish	-4.45	-7.93	**-12.3** *	-1.02	1.12	**13.3** *	0.19	-10.9	0.29
Red meat	-0.67	-3.57	1.35	0.25	-1.42	3.22	0.55	-7.45	-2.52
White meat	-6.64	**-10.8** *	**-11.4** *	0.09	-1.46	**-11.3** *	-6.28	-4.62	**-9.21** *
Processed meat	10.4	14.0	**17.5** *	-1.88	3.58	8.07	4.98	0.16	6.08
Liver	2.27	3.94	0.53	-0.48	0.97	-0.09	-2.76	-2.35	-1.26
Potatoes	-2.60	-2.04	-1.91	-0.11	0.40	-0.71	**4.26** **	1.82	**3.42** *
Cereal	0.46	1.58	0.11	**0.97** **	-0.96	2.00	0.21	-1.62	-0.41
Bread	1.80	0.47	**7.52** **	0.06	-0.28	-2.63	**-4.86** **	-2.53	**-3.38** *
Sweets	1.30	-0.07	3.16	-0.51	0.77	-2.91	-1.68	-0.27	-0.29
Nuts and seeds	0.73	1.64	-2.20	-0.19	0.16	1.27	**2.85** *	2.02	**2.53** *
Tea	0.09	-0.13	**1.31** **	0.13	-0.13	-0.55	-0.14	0.09	0.24
Coffee	-0.51	0.01	-0.66	-0.14	0.16	0.19	-0.09	0.14	0.45
Fruit juice	-1.41	-0.58	1.01	-0.62	**1.86** **	-1.97	-0.22	-3.21	0.15
Sugar-sweetened beverage	0.35	2.02	0.35	0.10	-0.09	0.11	0.96	1.15	1.21
Alcohol	**-5.98** **	-3.20	-1.96	**1.07** *	-1.41	-0.93	1.14	2.68	-0.35
Margarine	-1.08	-1.41 *	-0.99	-0.13	0.19	**-1.72** **	0.11	-0.97	-0.09
Vegetable oil	0.59	0.68	-0.01	-0.02	-0.06	**-2.16** **	**-1.36** *	-0.40	**-1.63** **

20 0 -20

Online Supporting Material

(B) Individual polyunsaturated fatty acids

Annual % changes (95%CI)	C20:5n3 2.89 (2.59, 3.2)	C22:5n3 0.93 (0.8, 1.06)	C22:6n3 0.83 (0.66, 0.99)	C18:2n6c -0.71 (-0.79, -0.63)	C18:3n6 0.97 (0.67, 1.27)	C20:2n6 -0.77 (-0.87, -0.67)	C20:3n6 0.39 (0.27, 0.51)	C20:4n6+ C20:3n3 0.67 (0.56, 0.77)	C22:4n6 -0.58 (-0.71, -0.44)	C22:5n6 0.01 (-0.18, 0.20)
Total Fruit	-0.72	-0.67	-1.07	0.51	1.11	0.64	-0.23	-0.66	-0.25	-0.29
Total vegetable	**4.52** *	1.20	**2.44** *	-0.15	-3.22	-0.63	-1.07	-0.82	**-2.07** *	-1.96
Legume	-5.21	-2.55	-3.76	-0.12	-1.49	-4.25	-3.22	-4.41	-4.45	-5.75
Total dairy	-0.22	0.23	-0.22	-0.42	1.17	0.74	**1.52** **	0.60	**1.36** *	1.53
Total eggs	-30.3	-0.91	5.80	0.31	30.1	-5.19	-4.79	**22.9** **	16.9	**40.3** **
White fish	-9.88	-7.18	-6.02	**9.96** *	-4.46	3.85	-5.67	-6.10	-8.20	-16.6
Fatty fish	**38.8** **	-4.22	**15.5** *	0.64	-2.20	-6.79	-9.26	**-10.2** *	**-17.5** **	**-19.2** **
Red meat	3.33	-2.57	4.00	-0.51	-12.4	-4.54	**-8.11** *	5.16	-4.63	-7.75
White meat	6.48	6.92	6.86	-0.27	2.99	2.84	4.44	0.54	-0.18	1.68
Processed meat	5.44	4.55	-3.23	-4.07	34.6	5.55	13.8	7.11	7.07	2.50
Liver	-4.88	0.67	-1.77	-1.16	4.82	**4.87** *	2.94	0.99	4.81	4.17
Potatoes	0.83	1.53	**-4.42** *	0.56	7.80	-1.33	1.06	-1.40	1.48	-1.90
Cereal	**-6.51** *	**-2.69** *	-3.18	1.30	-2.78	1.90	0.72	-1.50	-1.06	-1.22
Bread	-3.19	-1.30	-1.88	-0.36	7.61	**2.93** *	2.26	0.58	0.20	0.07
Sweets	6.61	-0.03	-0.19	0.20	-0.28	-1.08	-0.18	-1.82	-1.15	-2.41
Nuts and seeds	0.63	-2.36	0.08	0.61	-3.36	-0.48	**-2.67** *	0.76	-1.41	-0.22
Tea	0.04	0.46	-0.76	-0.19	0.92	-0.05	**0.77** *	-0.15	0.63	0.94
Coffee	1.17	**0.65** *	-0.03	-0.14	1.11	-0.07	0.41	-0.11	0.66	**0.94** *
Fruit juice	6.00	2.73	2.15	-0.40	-0.25	-2.07	0.34	0.13	-0.62	**-4.54** *
Sugar-sweetened beverage	-3.68	0.48	-0.99	-0.74	4.10	1.22	1.58	1.39	**3.29** **	**5.81** **
Alcohol	9.41	1.78	3.10	-0.29	0.22	1.66	-2.98	-0.98	-3.08	-4.81
Margarine	-0.74	-0.15	-0.86	0.70	2.18	-0.17	0.44	0.08	0.54	0.54
Vegetable oil	-1.43	-0.02	-0.71	0.64	0.09	0.29	-0.38	0.07	-0.28	0.19

(C) Individual monounsaturated fatty acids and trans fatty acids

Annual % changes (95%CI)	C16:1 0.92 (0.74, 1.1)	C17:1 -0.52 (-0.85, -0.18)	C18:1n9c 0.43 (0.34, 0.52)	C20:1n9 -1.77 (-1.98, -1.57)	C24:1 1.96 (1.80, 2.13)	C181:n9t -9.92 (-10.2, -9.65)	C18:2n6t -1.84 (-2.05, -1.64)
Total Fruit	-1.20	-1.55	0.18	-0.96	-0.52	-0.20	-0.18
Total vegetable	-0.33	**-5.01** *	-1.05	0.55	0.50	1.16	-1.44
Legume	0.56	6.58	-0.64	4.59	**-9.73** *	-9.18	9.29
Total dairy	0.75	-0.72	0.51	-0.09	0.88	0.11	1.11
Total eggs	5.43	-0.30	3.64	-23.8	5.38	40.0	-5.43
White fish	**-28.2** **	**-34.2** *	**-10.2** *	-17.8	4.98	13.2	-16.1
Fatty fish	**-15.1** *	27.7	-2.27	10.6	1.29	24.3	4.65
Red meat	-6.83	-5.63	-2.28	-9.93	1.75	8.98	-0.65
White meat	0.22	8.97	-2.88	**16.4** *	-4.59	14.6	-3.66
Processed meat	9.05	5.52	-0.05	-2.74	13.88	-24.6	-9.81
Liver	-0.16	0.31	2.68	-4.13	**-7.28** *	5.74	-5.72
Potatoes	-1.10	-2.95	1.04	3.64	2.30	-1.94	-2.53
Cereal	0.26	-3.64	0.39	-0.38	-0.21	**-6.15** *	-1.86
Bread	2.47	6.50	1.82	-0.73	-2.53	-0.91	**6.86** *
Sweets	0.69	3.98	0.13	**6.82** *	-2.75	1.13	1.00
Nuts and seeds	-2.39	-0.20	-1.16	-1.78	**3.20** *	-4.22	-1.93
Tea	0.29	-0.18	0.07	-0.76	-0.41	0.55	-0.68
Coffee	0.13	-1.14	**0.46** *	-0.64	-0.35	-0.09	-0.50
Fruit juice	-2.57	**1.0**	-1.37	3.26	-1.17	-6.97	**-5.16** *
Sugar-sweetened beverage	0.89	0.16	0.19	0.13	-0.06	**-4.60** *	-0.57
Alcohol	5.44	**14.2** *	0.97	-5.11	1.25	-2.40	-0.65
Margarine	-0.40	**-4.29** **	**-0.90** *	-0.57	**-1.86** **	1.13	0.70
Vegetable oil	0.01	3.13	-0.78	-0.34	**-1.87** **	-0.12	0.48

Supplemental Figure 3 Associations of baseline 23 food groups with annual percentage change in each plasma fatty acid group from 1993-1997 to 2004-2011. Mixed-effects linear regression models were used in the analyses. Each value represents an average relative difference (%) in changes/year in mol% of each fatty acid group from 1993-1997 to 2004-2011, per 1-standard serving/day higher in baseline food groups, with mutual adjustment for the baseline food groups, fatty acid change over the same time period and other potential confounders (* p<0.05 and ** p<0.01). Serving sizes were defined as 10g/d for margarine, liver, nuts and seeds; 1g/d for vegetable oil; 10 units/wk for alcohol and 100g/d for other food groups. Values in blue font represent negative associations; values in red font represent positive associations. SFA, saturated fatty acids; PUFA, polyunsaturated fatty acids; MFUA, monounsaturated fatty acids; TFA, trans fatty acids.

	Odd-chain SFA	Even-chain SFA	Very-long-chain SFA	Plant n-3 PUFA	Marine n-3 PUFA	n-6 PUFA	MUFA	TFA
Annual % changes (95%CI)	-0.63 (-0.73, -0.52)	-0.05 (-0.06, -0.03)	0.03 (-0.11, 0.16)	-0.23 (-0.51, 0.05)	1.28 (1.11, 1.44)	-0.25 (-0.29, -0.2)	0.45 (0.37, 0.54)	-7.84 (-8.07, -7.61)
Total Fruit	-0.03	0.00	-0.04	0.16	-0.01	-0.02	0.03	0.01
Total vegetable	0.07	-0.01	**0.20** **	-0.10	-0.02	0.02	-0.02	0.03
Legume	0.23	0.01	0.21	-0.70	**1.22** **	**-0.25** *	0.32	**-1.31** *
Total dairy	-0.05	0.01	-0.02	-0.07	0.04	-0.01	-0.04	0.07
Total eggs	-0.84	**0.16** *	0.10	1.50	**-2.10** **	0.39	-0.77	1.73
White fish	0.16	-0.04	-0.34	0.86	0.01	-0.23	0.16	1.91
Fatty fish	-0.37	-0.07	0.08	1.40	**-1.70** **	0.25	0.39	0.98
Red meat	0.05	0.04	-0.18	0.48	-0.17	0.04	-0.12	0.19
White meat	0.31	0.06	0.17	-0.55	0.14	**-0.28** *	**0.48** *	-0.12
Processed meat	-0.82	0.03	**-1.14** *	1.50	0.02	-0.02	0.26	-0.10
Liver	0.08	0.01	-0.06	-0.07	0.07	-0.10	0.22	0.36
Potatoes	0.07	-0.02	-0.26	-0.14	-0.32	0.09	-0.03	-0.01
Cereal	0.03	**-0.03** *	0.13	-0.03	0.09	0.01	-0.01	0.31
Bread	0.03	-0.02	0.09	0.02	0.07	-0.01	0.09	-0.03
Sweets	-0.05	0.01	-0.01	-0.02	0.01	0.02	-0.01	**-0.96** **
Nuts and seeds	-0.04	**0.04** **	-0.17	0.13	0.19	**-0.10** *	0.05	**0.06**
Tea	0.00	0.00	0.03	-0.03	0.00	-0.01	-0.01	0.03
Coffee	0.00	0.00	-0.01	0.01	-0.04	0.00	0.01	0.03
Fruit juice	0.09	-0.01	0.00	-0.04	-0.11	0.00	0.10	0.16
Sugar-sweetened beverage	0.04	0.00	0.07	-0.26	0.05	0.01	**-0.11** *	-0.09
Alcohol	0.15	**-0.04** **	0.00	-0.25	0.02	0.05	-0.04	**0.54** **
Margarine	0.05	0.00	-0.05	0.18	0.10	**-0.05** *	**0.12** **	**-0.26** **
Vegetable oil	0.08	0.00	0.06	-0.16	0.09	-0.05	0.07	-0.12

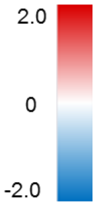

Supplemental Figure 4 Association between changes in 23 food groups from 1993-1997 to 1998-2000 and subsequent changes in each plasma fatty acid group from 1998-2000 to 2004-2011. Mixed-effects linear regression models were used in the analyses. Each value represents an average difference (%) in changes/year in mol% of each fatty acid group between 1998-2000 and 2004-2011, per annual 1-standard serving/day increase in each food group between 1993-1997 and 1998-2000, with mutual adjustment for changes in food groups and baseline consumption levels of those and adjusted for baseline levels of a fatty acid and other potential confounders (* p<0.05 and ** p<0.01). Serving sizes were defined as 10g/d for margarine, liver, nuts and seeds; 1g/d for vegetable oil; 10 units/wk for alcohol and 100g/d for other food groups. Values in blue font represent negative associations; values in red font represent positive associations. SFA, saturated fatty acids; PUFA, polyunsaturated fatty acids; MFUA, monounsaturated fatty acids; TFA, trans fatty acids.

	Odd-chain SFA	Even-chain SFA	Very-long-chain SFA	Plant n-3 PUFA	Marine n-3 PUFA	n-6 PUFA	MUFA	TFA
Annual % changes (95%CI)	-0.94 (-1.1, -0.78	-0.04 (-0.06, -0.02)	0.67 (0.5, 0.84)	-1.08 (-1.46, -0.7)	1.24 (1.01, 1.47)	-0.21 (-0.28, -0.14)	0.27 (0.15, 0.38)	-8.73 (-9.05, -8.4)
Total Fruit	0.09	-0.03	**0.55** *	-0.68	0.40	-0.03	0.02	0.35
Total vegetable	-0.14	0.01	**-0.92** **	-0.16	-0.16	0.06	0.26	-0.53
Legume	-0.74	0.14	2.25	0.22	-1.90	-0.06	0.01	4.45
Total dairy	0.09	0.00	0.30	-0.35	-0.23	0.05	-0.05	0.08
Total eggs	3.14	-0.03	-0.54	-5.21	0.58	-0.20	0.29	1.28
White fish	0.20	0.13	0.20	-3.99	-1.57	0.88	0.63	0.99
Fatty fish	0.24	0.21	-1.25	-6.16	-1.61	0.60	-0.78	-0.22
Red meat	0.40	-0.04	0.91	-0.55	2.06	-0.31	0.24	0.87
White meat	2.78	-0.15	0.65	-2.54	0.51	0.32	-0.73	-4.35
Processed meat	-3.19	0.20	-1.74	-2.36	-1.68	0.28	-0.84	-0.46
Liver	-1.90	-0.20	0.58	1.33	-0.89	0.66	-0.78	1.53
Potatoes	-0.29	-0.01	0.78	-0.25	0.19	0.14	-0.64	0.80
Cereal	-0.03	0.03	**-1.05** *	1.09	0.88	-0.20	0.16	-0.63
Bread	-0.67	-0.12	-0.35	0.15	-0.23	0.46	-0.37	1.31
Sweets	-0.34	0.10	0.69	0.69	-0.23	-0.15	-0.07	0.05
Nuts and seeds	0.31	-0.05	0.61	-0.90	0.34	-0.03	0.16	0.80
Tea	-0.02	-0.02	-0.09	0.00	0.12	-0.02	0.13	-0.02
Coffee	0.15	0.01	-0.05	-0.20	0.10	0.00	-0.13	-0.02
Fruit juice	0.36	0.03	0.35	0.59	-0.84	-0.11	0.59	**2.59** *
Sugar-sweetened beverage	-0.47	**0.14** **	-0.53	0.91	0.22	-0.27	0.00	0.06
Alcohol	-0.07	-0.11	-0.49	2.07	-0.67	0.18	0.33	0.62
Margarine	**0.44** *	0.04	0.02	-0.09	0.57	-0.11	-0.24	-0.15
Vegetable oil	-0.16	-0.01	0.09	0.29	-0.17	-0.03	0.28	0.08

Supplemental Figure 5 Association of changes in food subgroups with changes in plasma phospholipid fatty acid groups from 1993-1997 to 1998-2000. Mixed-effects linear regression models were used in the analyses. Values indicate an average relative difference in changes/year in mol% of each plasma fatty acid group between 1993-1997 to 1998-2000 per standard serving/day/year increase in each dairy or vegetable subgroup between the same time period (* p<0.05 and ** p<0.01). Serving sizes were defined as 100g/d for low-fat dairy, high-fat dairy, milk, yogurt, cheese, starchy and non-starchy vegetable; as 10g/d for butter, other dairy and avocado. Values in blue font represent negative associations; values in red font represent positive associations. Other dairy included single/double cream, dairy desserts (ex. dairy in other recipes. SFA, saturated fatty acids; PUFA, polyunsaturated fatty acids; MFUA, monounsaturated fatty acids; TFA, trans fatty acids.

	Odd-chain SFA	Even-chain SFA	Very-long-chain SFA	Plant n-3 PUFA	Marine n-3 PUFA	n-6 PUFA	MUFA	TFA
Low-fat dairy	**-1.11** *	0.08	0.22	0.26	-0.70	0.04	0.36	0.39
High-fat dairy	1.67	-0.13	-0.58	1.41	1.22	-0.15	0.20	-2.50
Milk	-0.98	0.00	-0.10	0.91	-0.51	0.08	0.57	0.11
Yogurt	1.84	**0.52** *	1.64	-6.71	-0.68	-0.22	-0.27	-5.73
Cheese	1.07	-0.95	**11.9** *	7.30	-2.44	-0.44	7.03	**-21.3** *
Other dairy	**1.47** *	0.10	-0.68	6.35	0.71	-0.99	1.42	-4.40
Butter	-2.00	-0.17	-0.51	0.64	1.44	-0.29	0.78	-0.30
Starchy vegetable	-2.10	0.14	**3.22** *	3.73	-2.01	-0.01	0.37	-4.74
Non-starchy vegetable	0.43	-0.07	-0.73	-1.38	2.50	-0.23	-0.98	0.95
Avocado	0.88	0.35	3.27	1.58	7.10	**-2.27** *	2.34	1.25

Color scale: 20, 0, -20

Supplemental Figure 6 Change of BMI and physical activity and with change of fatty acid groups. Mixed-effects linear regression models were used in the analyses. **(A)** represents an average relative difference (%) in changes/year in mol% of each fatty acid group, per 1 unit (kg/m^2) change in BMI between 1993-1997 to 1998-2000, adjusting for potential confounders; **(B)** represents an average relative difference (%) in changes/year in mol% of each fatty acid group, per 1-category change of physical activity status between 1993-1997 to 2004-2011, adjusting for potential confounders. SFA, saturated fatty acids; PUFA, polyunsaturated fatty acids; MFUA, monounsaturated fatty acids; TFA, trans fatty acids. *p=0.047 for the association between change in BMI and change in even-chain SFA.

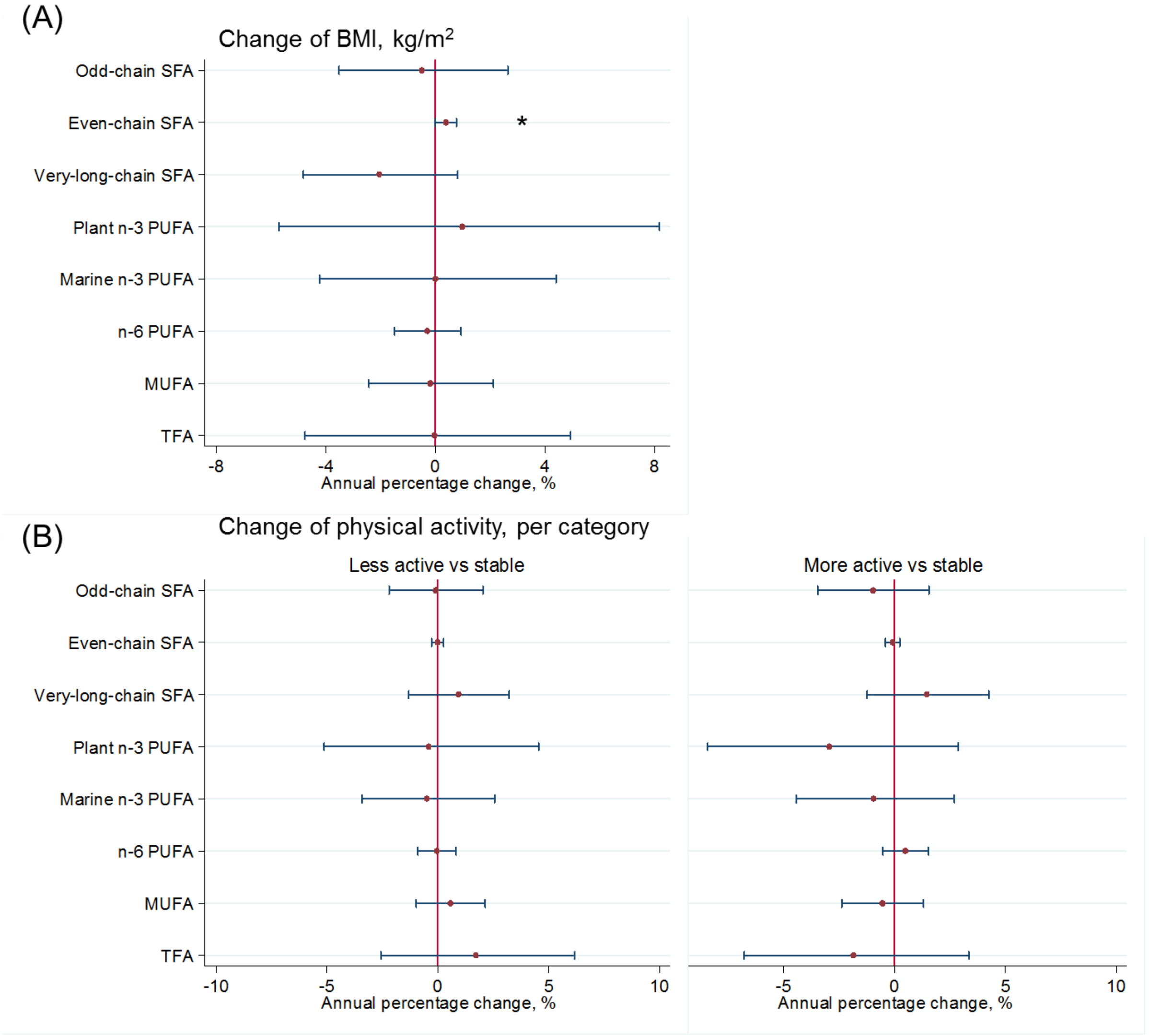

Supplement: nqz030_Supplemental_File [file nqz030_supplemental_file.pdf]
